# High-performance CRISPR-Cas12a genome editing for combinatorial genetic screening

**DOI:** 10.1038/s41467-020-17209-1

**Published:** 2020-07-13

**Authors:** Rodrigo A. Gier, Krista A. Budinich, Niklaus H. Evitt, Zhendong Cao, Elizabeth S. Freilich, Qingzhou Chen, Jun Qi, Yemin Lan, Rahul M. Kohli, Junwei Shi

**Affiliations:** 1 Department of Cancer Biology, Perelman School of Medicine, University of Pennsylvania, Philadelphia, PA 19104 USA; 2 Epigenetics Institute, Department of Cell and Developmental Biology, Perelman School of Medicine, University of Pennsylvania, Philadelphia, PA 19104 USA; 3 Abramson Family Cancer Research Institute, Perelman School of Medicine, University of Pennsylvania, Philadelphia, PA 19104 USA; 4 Department of Medicine, Perelman School of Medicine, University of Pennsylvania, Philadelphia, PA 19104 USA; 5 Department of Cancer Biology, Dana-Farber Cancer Institute, Department of Medicine, Harvard Medical School, Boston, MA 02215 USA; 6 Present Address: Department of Bioengineering, University of Pennsylvania, Philadelphia, PA 19104 USA

**Keywords:** Functional genomics, CRISPR-Cas systems, Genetic interaction

## Abstract

CRISPR-based genetic screening has revolutionized cancer drug target discovery, yet reliable, multiplex gene editing to reveal synergies between gene targets remains a major challenge. Here, we present a simple and robust CRISPR-Cas12a-based approach for combinatorial genetic screening in cancer cells. By engineering the CRISPR-AsCas12a system with key modifications to the Cas protein and its CRISPR RNA (crRNA), we can achieve high efficiency combinatorial genetic screening. We demonstrate the performance of our optimized AsCas12a (opAsCas12a) through double knockout screening against epigenetic regulators. This screen reveals synthetic sick interactions between *Brd9*&*Jmjd6*, *Kat6a*&*Jmjd6*, and *Brpf1*&*Jmjd6* in leukemia cells.

## Abstract (web summary)

Reliable, multiplexed gene editing to uncover synergies between targets remains challenging. Here, the authors engineer AsCas12a and the crRNA to improve double knockout for synthetic sick/lethal interaction genetic screening.

## Introduction

Combinatorial genetic screening, a high-throughput strategy to perturb multiple genes simultaneously, holds great promise for discovering therapeutically tractable synthetic sick/lethal interactions across diverse disease types, including cancers^1–7^. Currently, Cas9-based methods are efficient in single-gene knockout screening^8^ but greatly underperform in combinatorial genetic screening, primarily due to the complicated cloning and high recombination frequency of the dual CRISPR-Cas9 RNA (sgRNA) expression vector^9–12^. Cas12a (formerly Cpf1), an RNA-programmable DNA endonuclease similar to Cas9, has the potential to overcome these limitations. Unlike Cas9, Cas12a has intrinsic RNase activity that allows processing of its own crRNA array, enabling multigene editing from a single RNA transcript^13,14^ (Supplementary Fig. 1). This established characteristic of Cas12a makes it potentially suited to multiplex editing for combinatorial genetic screening. To date, however, Cas12a-based combinatorial genetic screening has only been performed in the setting of positive selection. No negative selection “dropout” screens have been reported, which is largely due to the low gene editing efficiency of Cas12a in mammalian cells^14,15^. Unlike in positive selection screens, where an external selection pressure facilitates nomination of hits, negative selection screens, or dropout screens, demand highly efficient CRISPR-Cas editing to increase signal, reduce noise, and successfully identify hits. In this work, we optimize the Cas12a system and demonstrate crRNA design principles for this optimized system. Together, these modifications achieve sufficient gene editing efficiency in mammalian cells for Cas12a-based dropout genetic screening. Subsequently, we apply this optimized Cas12a system in a double knockout screen to uncover leukemia-specific synthetic sick/lethal genetic interactions.

## Results

### AsCas12a and crRNA optimization increases mammalian editing

To unleash the intrinsic RNA-processing potential of Cas12a, we considered whether optimization of a Cas12a system could permit its use in combinatorial genetic screening. We selected *Acidaminococcus* Cas12a (AsCas12a) as an optimal starting point for engineering given its high gene editing activity in prior work^14^. However, the knockout efficiency of AsCas12a in human cells is significantly lower than that of *Streptococcus pyogenes* Cas9 (SpCas9)^14^. Given that purified AsCas12a was as active as SpCas9 in an in vitro editing assay^14^, we hypothesized that suboptimal nuclear localization might be partially responsible for the low knockout efficiency of AsCas12a. Inspection of the amino acid sequence revealed that there are two potentially problematic nuclear export signals (NESs) in AsCas12a, including one in the highly conserved catalytic RuvC-II domain (Supplementary Fig. 2). In support of this possibility, we found that both increasing the number of either N- or C-terminal nuclear localization signals (NLSs) and replacing the common SV40 NLS with the c-Myc NLS improved the knockout efficiency of AsCas12a (Supplementary Fig. 3).

**Fig. 1:**
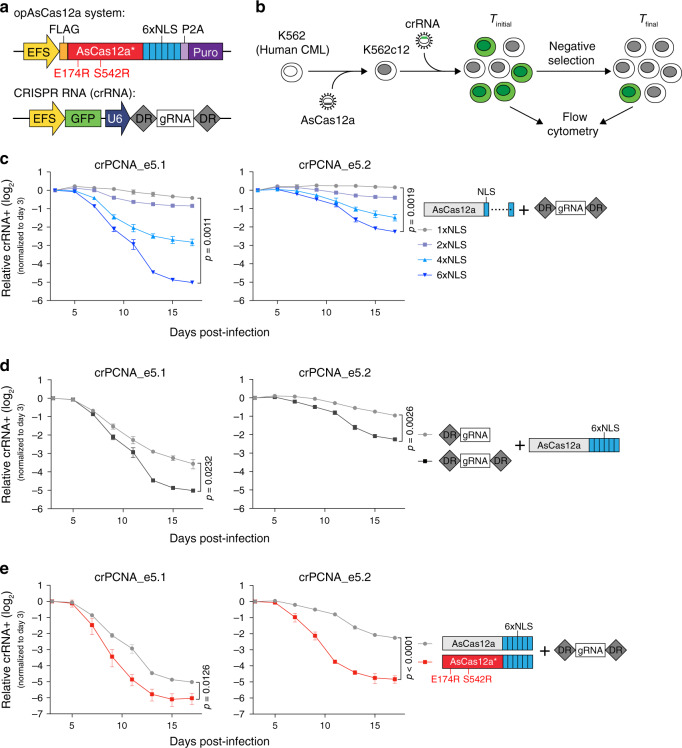
Optimization of an AsCas12a system to improve knockout efficiency in mammalian cells. **a** Configuration of the optimized vectors used for lentiviral AsCas12a and CRISPR RNA (crRNA) transduction experiments in mammalian cells. A Puromycin (Puro) resistance gene was used to select AsCas12a-positive cells, and a GFP reporter was linked with crRNA expression to track the crRNA-positive population in cellular competition assays. **b** Experimental workflow of cellular competition assay to evaluate AsCas12a knockout efficiency. K562 cells were stably transduced with indicated AsCas12a vector, followed by secondary infection with crRNA virus. Flow cytometry-based tracking of the crRNA-positive cell population over a period of time was used to calculate the negative selection phenotype of each indicated crRNA. Cellular competition assay to compare the knockout efficiency of the AsCas12a system with a variable number of NLS sequences (**c**), with or without an additional direct repeat (DR) 3′ of the crRNA (**d**), and with an AsCas12a variant (E174R and S542R) (**e**). Plotted are the crRNA-positive cells (normalized to the day 3 measurement) at the indicated timepoints during culturing. Two crRNAs were designed targeting *PCNA*, an essential gene for DNA replication. “e” represents the exon. (*n* = 3). *p* values are indicated (two-tailed Welch’s unpaired *t*-test). Error bars smaller than symbol width not shown. All error bars shown represent mean ± SD. Source data are provided as a Source Data file.

Deep sequencing-coupled pooled genetic screens require single-copy viral transduction, in which both AsCas12a protein and crRNA expression are greatly reduced compared to transient transfection. To increase the knockout efficiency of AsCas12a and make it permissive for dropout genetic screening, we next subjected the system to three levels of engineering. In order to evaluate the knockout efficiency of our variants in this system, we expressed AsCas12a and its crRNA using a two-vector lentiviral expression strategy and conducted a competition-based cellular proliferation assay, a reliable proxy for dropout screening (Fig. 1a, b). We designed crRNAs targeting known essential genes, including replication protein *PCNA* and cyclin-dependent kinase *CDK1*. We assessed the AsCas12a knockout efficiency by measuring the negative selection strength of crRNAs targeting these essential genes in a 17-day time-course. First, to counter the effect of the NESs, we found that introducing more NLSs significantly increased AsCas12a knockout efficiency in K562 cells, with a dramatic 4- to 32-fold improvement noted with the addition of six NLSs (Fig. 1c and Supplementary Fig. 4). It has been previously reported that additional hairpin structures 3′ of SpCas9 sgRNA could improve sgRNA stability and thus gene editing^16^, potentially by protection from 3′-exonuclease degradation. We therefore explored adding a direct repeat (DR) 3′ of the AsCas12a crRNA and found that this change further enhanced knockout efficiency by approximately twofold (Fig. 1d and Supplementary Fig. 4). Finally, we introduced the E174R and S542R mutations postulated to increase DNA binding affinity and activity^17^, termed AsCas12a* here, into our AsCas12a-6xNLS. Combined, our optimizations improved knockout efficiency by ~32- to 64-fold total over the original baseline (Fig. 1c, e). We also tested our optimized AsCas12a*-6xNLS with nontargeting dual-DR crRNAs and dual-DR crRNAs targeting introns of *PCNA* and *CDK1* in K562 cells. We observed no measurable effects on cellular proliferation, suggesting that our final optimized system was not toxic to the cells (Supplementary Fig. 5). Together, our investigation of various modifications establishes that the combination of 6xNLS, dual-DR crRNA, and AsCas12a* protein greatly enhances knockout efficiency (this optimized AsCas12a* and crRNA system was named opAsCas12a) (Fig. 1a).

### opAsCas12a performs robustly in single-gene knockout screens

**Fig. 2:**
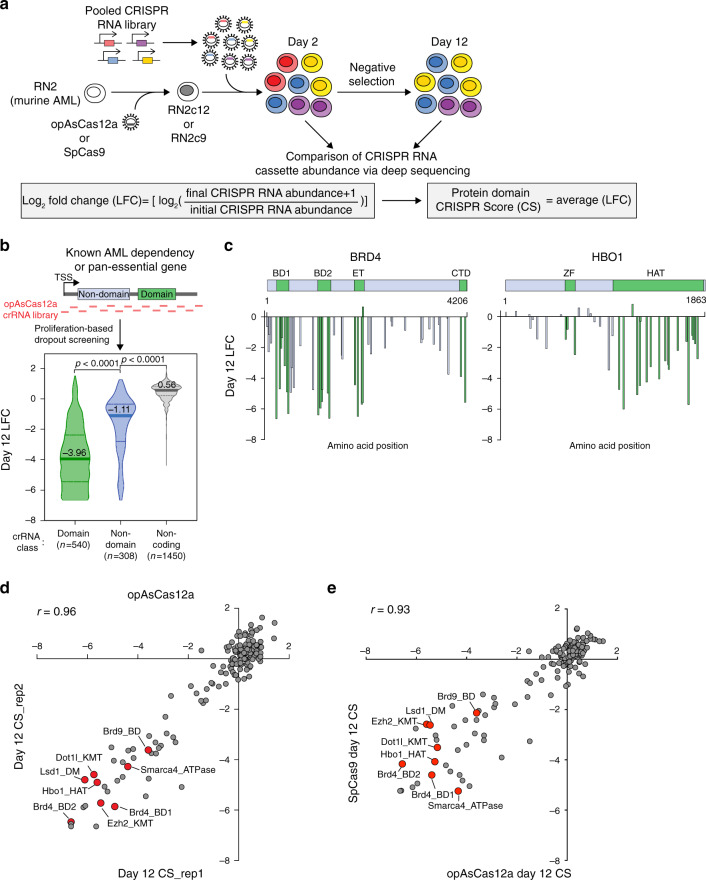
opAsCas12a performs similarly to SpCas9 in domain-focused single-gene knockout screens. **a** Experimental schematic of pooled dropout screen. See “Methods” for detailed explanation. A protein domain CRISPR Score (CS) was calculated by averaging the log_2_ fold-change of all CRISPR RNA targeting a given protein domain. Fold-change = (final CRISPR RNA abundance + 1)/(initial CRISPR RNA abundance). **b** Comparative functional knockout efficiency of AsCas12a crRNAs targeting conserved protein domains, nondomain coding regions, and noncoding regions of known AML dependencies and pan-essential genes. RN2c12 cells were lentivirally transduced with a customized pooled library containing 2298 total crRNAs (*n* indicates the number of crRNA in each crRNA class). Violin plot demonstrates median (thick line, value listed), interquartile (dashed lines), and distribution of guide-wise log_2_ fold-change of two replicate screens. *p* values are indicated (Two-tailed Mann–Whitney *U*-test). **c** Individual examples of functional protein domain-targeting crRNAs that lead to a higher proportion of null mutations and an enhanced severity of dropout. The location of each crRNA cleavage site within the BRD4 and HBO1 coding sequences is indicated along the *x*-axis. Plotted is the mean log_2_ fold-change value of two biological replicates (*n* = 2). crRNAs targeting inside or outside of conserved protein domains are represented by green or blue bars, respectively. BD=bromodomain, ET=extraterminal domain, CTD= C-terminal domain, ZF=zinc finger, HAT=histone acetyltransferase. **d** The result of pooled opAsCas12a dropout screen to identify epigenetic dependencies in RN2c12 cells. A customized opAsCas12a crRNA library against 155 protein domains involved in epigenetic regulation was constructed. crRNAs were designed to target epigenetic regulatory protein domains^18^, yielding 787 crRNAs total including positive and negative controls. RN2c12 or RN2c9 cells were transduced with pooled opAsCas12a crRNA or SpCas9 sgRNA libraries. Scatter plot that compares the CSs of two independent replicates (*r* = 0.96, Pearson correlation). **e** Comparison of opAsCas12 and SpCas9 in pooled dropout screen against epigenetic regulators in RN2 cells. Plotted is the average CS of two biological replicates (*n* = 2). (*r* = 0.93, Pearson correlation). **d**, **e** Red dots label known cancer drug targets in this Mll-Af9 leukemia model. Source data are provided as a Source Data file.

To benchmark the performance of our opAsCas12a system against SpCas9, we conducted a single-gene dropout screen to identify known cancer drug targets. We performed the opAsCas12a- and SpCas9-based screens in a murine Mll-Af9/Nras^G12D^ acute myeloid leukemia cell line (RN2), which has previously been used to identify epigenetic dependencies with SpCas9^18^. To start, we created an opAsCas12a-expressing RN2 cell line (RN2c12) (Fig. 2a). We then verified the high knockout efficiency of our opAsCas12a system using crRNAs targeting essential gene *Rpa3* and known leukemia dependency *Brd4*^19^ (Supplementary Fig. 6). Previously, we found that sgRNA targeting conserved protein domain regions generate a higher proportion of functional knockout alleles than sgRNA targeting early exon sequences, thus improving the robustness of Cas9-based genetic screens^18^. To determine how the AsCas12a target position within a protein-coding gene affects functional knockout generation, we designed a crRNA library that spans the coding and noncoding regions of a set of known leukemia dependencies and pan-essential genes (Fig. 2b). These screen results validated that crRNAs targeting conserved protein domains showed stronger dropout phenotypes than crRNAs targeting nondomain exonic regions. crRNAs targeting noncoding regions had minimal effect on cellular proliferation (Fig. 2b, c and Supplementary Figs. 7 and 8).

Based on the domain-targeting rule, we constructed a customized opAsCas12a crRNA library targeting 155 protein domains involved in epigenetic regulation. We next performed dropout screens in RN2c12 cells with this epigenetic-focused opAsCas12a crRNA library. For comparison, we also performed screens using either wildtype (WT) AsCas12a (6xNLS and dual-DR) or our previously published SpCas9 sgRNA library^18^ (Fig. 2a, d, e and Supplementary Figs. 9 and 10). A log_2_ fold-change (LFC) value was calculated for each CRISPR RNA and a CRISPR Score (CS), defined as the average LFC of all CRISPR RNAs targeting a given protein domain, was used to quantify each domain’s essentiality in supporting cancer cell proliferation (Fig. 2a). Spike-in positive and negative control CRISPR RNAs included in the libraries validated the overall accuracy of all three pooled screens (Supplementary Figs. 10 and 11). At the level of individual CRISPR RNA, LFCs of opAsCas12a screens were highly correlated between biological replicates (*r* = 0.87) and suggested similar robustness to the SpCas9 screen data (*r* = 0.89). At the protein domain level, CSs of opAsCas12a screens were highly correlated between biological replicates (*r* = 0.96) and with the SpCas9 screen data (*r* = 0.93) (Fig. 2d, e). WT AsCas12a CSs demonstrated lower precision between replicate screens (*r* = 0.85) than either opAsCas12a or SpCas9 and did not correlate as well with SpCas9 CSs (*r* = 0.81) (Supplementary Fig. 9). Notably, all three screens revealed all known drug targets and dependencies in this murine Mll-Af9 leukemia model^18–26^ (Fig. 2d, e and Supplementary Fig. 9). Collectively, these data highlight the strength of a domain-focused analysis of protein-coding gene function and support our conclusion that our opAsCas12a system performs with similar robustness to SpCas9 in single-gene dropout screening.

### opAsCas12a pairwise screens find synthetic sick interactions

While single-gene knockout screens can capture a small subset of potential cancer drug targets, they miss synthetic sick/lethal genetic interactions that arise only from the simultaneous perturbation of two or more genes, representing a significant gap in the current genetic screening toolbox. We next sought to take advantage of the multigene knockout capacity of opAsCas12a for combinatorial genetic screening. To evaluate the double knockout efficiency of opAsCas12a and explore the generalizability of our approach, we performed cellular competition assays in a collection of seven diverse murine and human cell lines by expressing rearranged dual-crRNAs targeting essential genes and negative control loci under one U6 promoter (Supplementary Figs. 12 and 13). We confirmed that opAsCas12a can achieve targeted gene knockout effectively using a dual-crRNA expression array. We also observed that the phenotypic effect of a crRNA was biased slightly in favor of the first position 3′ to the U6 promoter in five out of seven cell lines tested (Supplementary Figs. 12 and 13).

The high recombination frequency of DNA elements in a Cas9 dual-sgRNA expression vector largely restricts its application in combinatorial genetic screens^9–12^. This recombination, which uncouples paired sgRNA-encoding DNA elements, can occur during lentiviral production, PCR amplification, and deep sequencing. Others have reported 10–40% sgRNA uncoupling in Cas9 systems^9–12^. We predicted that the recombination frequency would be significantly lower for AsCas12a, since the length of homologous sequence between two crRNAs is only 19–20 nt, compared to ~194–352 nt in Cas9-based dual-sgRNA systems (Supplementary Fig. 14a). To measure the DNA uncoupling frequency in our opAsCas12a system, we performed a mock genetic screen by subjecting a library of 168 unique dual-crRNAs to lentiviral production, PCR amplification, and deep sequencing. We found a DNA uncoupling frequency of ~0.33% in the opAsCas12a dual-crRNA cassette (Supplementary Fig. 14b), ~30 times lower than that observed with Cas9-based systems.

**Fig. 3:**
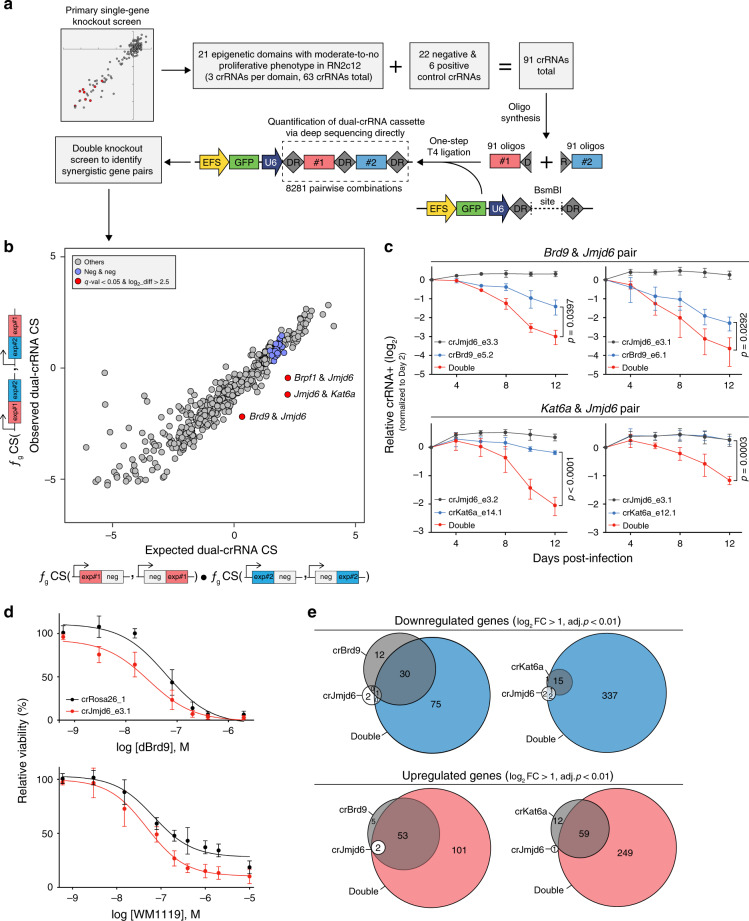
opAsCas12-based combinatorial knockout screen in Mll-Af9 leukemia identifies synthetic sick/lethal interaction of epigenetic regulators. **a** Cloning strategy to construct pairwise combinatorial AsCas12a dual-crRNA library. Experimental workflow to construct a customized dual-crRNA library. Twenty-one domains of epigenetic regulators with moderate-to-no proliferative phenotype in RN2 cells were selected from the single-gene knockout screens performed in Fig. 2. A dual-crRNA library with 8281 pairwise combinations was generated using a simple one-step T4 ligation protocol. The screen was performed in RN2c12 cells, and the abundance of crRNA pairs was quantified by directly deep sequencing the dual-crRNA expression cassette. **b** An opAsCas12a-based double knockout screen reveals synthetic sick/lethal epigenetic regulator pairs in RN2c12 cells. Scatter plot compares the expected and observed dual-crRNA CSs. The expected dual-crRNA CS was calculated based on a Gaussian distribution model of the experimental crRNAs in all possible combinations with negative control crRNAs. The observed dual-crRNA CS was calculated based on a Gaussian distribution model of all crRNA combinations of the two experimental protein domains. Blue dots represent the [negative crRNA]–[negative crRNA] control pairs and red dots represent potential synthetic sick/lethal genetic interactions (log_2_ differential > 2.5, *q*val < 0.05). **c** Cellular competition assay in RN2c12 cells to validate the screen identified potential synergistic interacting dual-crRNAs. Plotted are the dual-crRNA-positive cells (normalized to the day 2 measurement) at the indicated timepoints during culturing. Each data point is comprised of pairwise combinations of the two indicated crRNAs. *p* values are indicated (two-tailed Welch’s unpaired *t*-test). (*n* = 3). **d** Viability of RN2c12 cells transduced with either *Jmjd6* or *Rosa26* crRNA and exposed to increasing concentrations of either dBrd9 or WM-1119 for 5 days. dBrd9 is a selective BRD9 degrader and WM-1119 is a KAT6A/B inhibitor. (*n* = 3–5). **e** Venn diagram of RNA-seq data depicting the overlap of significantly upregulated and downregulated genes upon double knockout with indicated dual-crRNAs in RN2c12 cells. The second crRNA in the dual-crRNA cassette for single-crRNA samples targets the negative control *Rosa26* locus. Error bars smaller than symbol width not shown. All error bars shown represent mean ± SD. Source data are provided as a Source Data file.

Next, we aimed to evaluate how well opAsCas12a identifies synthetic sick/lethal genetic interactions in double knockout dropout screening. Since the most surprising synthetic sick/lethal interactions are those entirely undetectable unless combined with other knockouts, genes with moderate-to-no knockout phenotype on their own provide the best opportunity to reveal unexpected interactions. We selected 21 epigenetic regulatory domains with moderate-to-no effect on proliferation from the single-crRNA opAsCas12a screen (Fig. 2d). While a single-gene dropout screen would be unable to identify a proliferation phenotype associated with such genes, a combinatorial screen can reveal synthetic sick/lethal interactions caused by the double knockout of genes with moderate-to-no individual phenotype. We then constructed a dual-crRNA library with 8281 pairwise combinations using a simple one-step T4 ligation approach (Fig. 3a). We performed the screen in RN2c12 cells and quantified the abundance of crRNA pairs by directly deep sequencing the dual-crRNA expression cassette. As in the cellular competition assay (Supplementary Figs. 12 and 13), we observed an apparent crRNA position effect during pooled library screening, where 75% of the experimental crRNA showed a stronger effect in the first position of a dual-crRNA array (Supplementary Fig. 15). To account for this position bias, a Gaussian distribution model was used to adjust the dual-crRNA CS (ƒ_g_CS). When an observed ƒ_g_CS of two protein domains is significantly, at least two standard deviations, lower than the expected score from the combination of individual-domain ƒ_g_CS, this domain pair is considered to have a synergistic interaction (see supplementary methods for detailed explanation). Interestingly, our double knockout screen identified three synthetic sick interaction pairs, *Brd9*&*Jmjd6*, *Kat6a*&*Jmjd6*, and *Brpf1*&*Jmjd6*, which, when perturbed simultaneously, showed stronger proliferation inhibition than when each gene was targeted individually (Fig. 3b). *Jmjd6* appeared in all three pairs, suggesting that its perturbation might sensitize RN2 cells to various stresses.

BRD9 participates in nucleosome remodeling, JMJD6 functions as a protein hydroxylase or histone demethylase, and KAT6A acts as a histone acetyltransferase. Based on their known functions, it would not be obvious to predict synergistic interaction of any of these three genes in leukemia. We proceeded to validate the *Brd9*&*Jmjd6* and *Kat6a*&*Jmjd6* interactions because small molecule inhibitors of BRD9 and KAT6A are available^27,28^. We first validated the on-target gene editing effects of *Brd9*&*Jmjd6* and *Kat6a*&*Jmjd6* dual-crRNAs in RN2c12 and B16-F10c12 melanoma cells (Supplementary Fig. 16). Then, using cellular competition assays, we confirmed that double knockout of either *Brd9*&*Jmjd6* or *Kat6a*&*Jmjd6* had a synergistic suppressive effect on RN2 proliferation, but had minimal-to-no effect on both NIH3T3c12 fibroblast and B16-F10c12 cellular proliferation, suggesting that these two screen-identified synthetic sick/lethal genetic interactions might be leukemia-specific (Fig. 3c and Supplementary Figs. 16 and 17). Next, we used chemical inhibitors to further validate these two screen-identified hits. Since *Jmjd6* crRNAs had a minimal effect on cellular proliferation of RN2c12 cells (Fig. 3c), we generated a stable *Jmjd6*-deficient cell line and tested its sensitivity to either a BRD9 or KAT6A/B inhibitor. Consistent with the dual-crRNA perturbation data, *Jmjd6* knockout sensitized RN2c12 cells to both BRD9 and KAT6A/B inhibitors (Fig. 3d). To further characterize the role of *Brd9*&*Jmjd6* and *Kat6a*&*Jmjd6* in leukemia, we performed RNA-seq to compare the global transcriptional changes upon either single or double genetic perturbation of the targeted genes. This analysis suggested that both double knockout of *Brd9*&*Jmjd6* and *Kat6a*&*Jmjd6* have a cooperative effect on transcription when compared to either single knockout (Fig. 3e and Supplementary Fig. 18a). Compared to either single knockout, we observed that double knockout of *Brd9*&*Jmjd6* and *Kat6a*&*Jmjd6* were associated with stronger transcriptional changes of two gene sets linked to leukemia stem cell and myeloid differentiation programs, both of which are essential regulatory programs for leukemia survival^20^ (Supplementary Fig. 18b). Together, our data demonstrate the robustness of our opAsCas12a system in combinatorial genetic screening for identifying synergistic genetic interactions.

## Discussion

In this work, we optimized gene knockout efficiency of CRISPR-AsCas12a in mammalian cells by modifying both the Cas protein and the crRNA, resulting in orders of magnitude increase in efficiency. These modifications permit the use of AsCas12a to conduct combinatorial dropout genetic screening with high efficiency by harnessing Cas12a’s intrinsic strength for multiplexing. We showed that construction of an opAsCas12a dual-crRNA library requires only a simple one-step T4 ligation cloning reaction with low recombination frequency, making Cas12a-based combinatorial genetic screening more easily accessible than a Cas9-based approach. Through double knockout screening, we have identified and validated nonobvious synergistic epigenetic vulnerabilities in Mll-Af9 leukemia cells, demonstrating the power of this method. During the preparation of this manuscript, an independent study led by John Doench and colleagues reported similar conclusions (10.1038/s41587-020-0600-6). The high performance of this new generation of AsCas12a-based approaches will unleash the next wave of combinatorial genetic screens across cancer types and disease models for therapeutic target discovery.

## Methods

### Cell lines

RN2, a murine Mll-Af9 cell line, was derived from an Mll-Af9 knock-in mouse^29^. RN2, and its derivative cell lines, were cultured in RPMI-1640 supplemented with 10% FBS. HEK293T (ATCC Cat# CRL-3216, RRID:CVCL_0063), NIH3T3 (ATCC Cat# CRL-1658, RRID:CVCL_0594), B16-F10 (ATCC Cat# CRL-6475, RRID:CVCL_0159), and A549 (ATCC Cat# CCL-185, RRID:CVCL_0023) cells were cultured in DMEM supplemented with 10% bovine calf serum. K562 (ATCC Cat# CCL-243, RRID:CVCL_0004), MOLM13 (DSMZ Cat# ACC-554, RRID:CVCL_2119), and HEL (ATCC Cat# TIB-180, RRID:CVCL_2481) cells were cultured in RPMI-1640 supplemented with 10% bovine calf serum. All cell culture media was supplemented with 1% penicillin/streptomycin. All cell lines were cultured at 37 °C with 5% CO_2_ and were periodically tested to be mycoplasma-negative.

### Vector construction and crRNA cloning

The protein-coding sequence of AsCas12a (RRID:Addgene_84739) was subcloned into a lentiviral expression vector, EFS-FLAG-P2A-Puro (RRID:Addgene_108100). NLS sequences were incorporated into overlapping DNA oligonucleotides homologous to the Cas-containing backbone by PCR. AsCas12a (E174R, S542R) was cloned in two rounds of mutagenesis PCR on the completed WT AsCas12a-6xNLS vector to generate a final lentiviral vector, opAsCas12a (pRG232, Addgene_149723). All vector cloning was performed using the In-Fusion HD Cloning system (TBUSA).

The AsCas12a crRNA expression vector (pRG212, Addgene_149722) was built by replacing the sgRNA region in the CROPseq-Guide-Puro plasmid (RRID:Addgene_86708) with AsCas12a DRs flanking a short filler that contained BsmbI restriction sites. In addition, the puromycin resistance cassette was replaced with EGFP-P2A-Neomycin, subcloned from LRG2.1-Neomycin (RRID:Addgene_125593).

The resulting pRG212 (EFS-GFP-P2A-Neo-U6-crRNA) vector was BsmbI-digested, and crRNAs were ligated in with T4 DNA ligase (NEB). Single-crRNA and dual-crRNA were cloned by annealing and phosphorylating complementary DNA oligonucleotides with T4 polynucleotide kinase (NEB).

### Lentivirus transduction

Lentivirus was produced by transfecting HEK293T cells with helper plasmids VSVG and psPAX2 (RRID:Addgene_12260) using polyethylenimine (Polysciences, PEI 25000) in a mass ratio of 4:2:3 for plasmid DNA:VSVG:psPAX2. Media was replaced ~6–8 h post transfection, and viral supernatant was collected several times within 24–72 h of transfection. Supernatant was passed through a 0.45 µm PVDF filter before use (Millipore). Lentivirus was added to target cell lines with 8 µg/mL Polybrene (Sigma #H9268) and centrifuged at 650 × *g* for 25 min at room temperature. Media was changed 15 h post infection. Antibiotics (1 µg/mL puromycin and/or 50 mg/mL G418) were added 15 h post infection when selection was needed.

### GFP disruption assay

HEK293T cells were first lentivirally transduced with a destabilized GFP (d2GFP) reporter (derived from Addgene #14760). The resulting HEK293T d2GFP reporter line was then transiently transfected with indicated AsCas12a vector and a vector expressing GFP-targeting crRNA. This d2GFP HEK293T reporter line was used to evaluate the knockout efficiency of seven Addgene-available Cas12a systems (*Butyrivibrio sp*., *Thiomicrospira sp*. *XS5*, *Moraxella bovoculi*, *Prevotella bryantii*, *Bacteroidetes oral*, *Lachnospiraceae bacterium*, and *Acidaminococcus*). In addition, AsCas12a vector contained 1, 2, 4, or 6 SV40 or c-Myc NLS cloned into either the N- or C-terminus. The crRNA vector contained an expression marker for mCherry for successful transfection tracking. Cells were cultured for 3 days and subjected to flow cytometry to measure knockdown of GFP using the Guava Easycyte 10 HT instrument (Millipore). Percent GFP disruption is plotted. Two independent GFP-targeting crRNA are presented.

### Protein extraction and western blot

Approximately 5 × 10^6^ K562-AsCas12a-xNLS cells were collected for nuclear extraction. After an initial PBS wash, nuclear protein fractions were isolated with the Compartment Protein Extraction Kit (Millipore, Catalog #2145) according to the manufacturer’s instructions. To prepare whole cell lysates, 2 × 10^6^ cells were resuspended in Laemmli sample buffer (Bio-Rad) containing 5% β-mercaptoethanol, and then denatured at 95 °C for 10 min. The protein extracts were separated on a 4–15% TGX gel (Bio-Rad), transferred to a nitrocellulose membrane, and analyzed by immunoblotting. All primary antibodies used at 1:1000 dilutions: FLAG (Sigma-Aldrich Cat# F1804, RRID:AB_262044), Lamin-B1 (Abcam Cat# ab16048, RRID:AB_10107828), and α-Tubulin (Sigma-Aldrich Cat# T6199, RRID:AB_477583). Secondary antibodies used: anti-Rabbit (LI-COR Biosciences Cat# 925-32211, RRID:AB_2651127), anti-Mouse (Thermo Fisher Scientific Cat# A-21058, RRID:AB_2535724). Blots were imaged on a Licor Odyssey. Relative abundance of protein was quantified via gel densitometry with ImageJ software. AsCas12a protein levels in the nuclear fraction were first normalized to Lamin-B1 levels and then to the corresponding 1x NLS samples.

### Competition-based cellular proliferation assays

Indicated AsCas12a-expressing cell lines were infected with a lentivirus (pRG212) encoding crRNA, targeting essential genes or known cancer dependencies, linked to a GFP reporter, to track successful transduction, at a multiplicity of infection (MOI) of around 0.2–0.5. The percentage of crRNA-positive (GFP-positive) cell population was monitored over time using the Guava Easycyte 10 HT instrument (Millipore). To assess the impact of a specific single-crRNA or dual-crRNA on cellular proliferation, final time point GFP% was divided by initial time point GFP% to calculate the negative selection fold-change of the crRNA-positive cell population.

### Epigenetic regulator single-gene knockout screening

Overall, 23-nt crRNA sequences were designed in Benchling to target functional protein domains using the protospacer adjacent motif (PAM) 5′-TTTV. The AsCas12a library gene list was adapted from a previously published SpCas9 pooled genetic screening against epigenetic regulators^18^. Approximately five crRNAs were designed for each domain, though some domains contain fewer (≥3 crRNAs) due to limited TTTV PAM abundance. An oligonucleotide pool containing the crRNA sequences flanked by BsmbI recognition sites and 24-nt primer sites^30^ was synthesized (Twist Bioscience). The pool was amplified with Phusion Hot Start Flex DNA Polymerase (NEB) over 20 cycles with an annealing temperature of 65 °C. The PCR product was gel-purified (Macherey-Nagel) and ligated into the BsmbI-digested pRG212 backbone using Golden Gate cloning. The ligation product was electroporated into MegaX DH10B electrocompetent cells (Invitrogen) with an ECM 630 Electroporation System (BTX) according to the manufacturer’s protocol. The cloned crRNA library was cultured overnight at 30 °C to minimize recombination, and the DNA was extracted with the PureLink HiPure Plasmid Maxiprep Kit (Invitrogen). Diversity of crRNA abundance was verified by Sanger sequencing randomly-picked bacterial colonies. To confirm that all designed crRNAs were cloned into the pRG212 vector, deep sequencing analysis was performed on either a MiSeq or NextSeq instrument (Illumina). The design of the single-gene AsCas12a crRNA library is provided (Supplementary Data 3).

Single-gene knockout screening using either SpCas9, WT AsCas12a, or opAsCas12a was performed in murine Mll-Af9/Nras^G12D^ acute myeloid leukemia cell line, RN2, stably expressing the corresponding Cas protein. The RN2c9 cell line was generated as a serial dilution-derived SpCas9 positive clonal line^18^. Lentivirus containing the pooled library was prepared and used to infect cells as described above. The lentivirus was titrated to achieve an MOI of ~0.3–0.5 to ensure single-copy viral integration, and cells were maintained at exponential growth. Cells were harvested on day 2 post infection as a reference for the fully represented pooled CRISPR RNA library. Cells were then cultured for 10 additional days and harvested on day 12 post infection. Either AsCas12a crRNA or SpCas9 sgRNA positive cells were cultured at ≥1000x CRISPR RNA coverage to maintain full representation of the pooled CRISPR RNA library. Harvested cells were washed in PBS, pelleted, and stored at −80 °C until genomic DNA extraction. Genomic DNA was isolated from the stated initial and final timepoints, and CRISPR RNA cassette quantification was conducted through deep sequencing.

### Design principle single-gene knockout screening

Overall, 23-nt crRNA sequences were designed in Benchling to target the functional protein domains, nondomain coding regions, and noncoding regions of pan-essential and leukemia-essential genes using variable PAMs 5′-TTTV (TTTA, TTTC, or TTTG). On average, 40 crRNAs were designed per gene. Domain crRNAs target Pfam- or UniProt-identified domain regions of NCBI RefSeq exons, determined using the UCSC genome browser. Nondomain crRNAs target nondomain exonic regions of NCBI RefSeq genes. Noncoding crRNAs target intronic or untranslated regions of NCBI RefSeq genes. crRNAs with a perfect match to any off-target sites in the genome were filtered out. The final 2298 AsCas12a crRNA list for analysis is included in Supplementary Data 2. An oligonucleotide pool containing crRNA sequences was ligated into the crRNA vector according to the aforementioned single-gene knockout screening protocol. To confirm that all designed crRNAs were cloned into the pRG212 vector, deep sequencing analysis was performed on a MiSeq instrument (Illumina).

Single-gene knockout screening using opAsCas12a was performed in RN2 cells stably expressing the Cas protein. Lentivirus containing the pooled library was prepared and used to infect cells as described above. Cells were harvested on day 2 post infection as a reference for the fully represented pooled CRISPR RNA library. Cells were then cultured for 6, 8, and 10 additional days and harvested. crRNA-positive cells were cultured at ≥×1000 crRNA coverage to maintain full representation of the pooled crRNA library. Harvested cells were washed in PBS, pelleted, and stored at −80 °C until genomic DNA extraction.

### Double knockout screening

Twenty-one domains of epigenetic regulators with moderate-to-no proliferation phenotypic effect in RN2 cells were adapted from the single-gene knockout screens performed in Fig. 2 (Supplementary Data 4). Three crRNAs from the single-gene knockout screening were selected for each gene. Separate oligonucleotide pairs were ordered for each crRNA for the first and second positions in the dual-crRNA expression vector. The oligonucleotide pairs were all phosphorylated and annealed separately, before being pooled together with the BsmbI-digested pRG212 backbone in a ligation reaction (T4 DNA ligase). The ligated product was electroporated into the MegaX DH10B cells. Lentivirus production of the pooled library, infection of the opAsCas12a-transduced RN2 cells, and the screening itself were carried out as in the single-gene knockout screening described above.

### Deep sequencing library construction

Genomic DNA was isolated from the screening samples collected at the initial and final timepoints with the Quick-DNA Mini or Midiprep Plus kit (Zymo Research) following the manufacturer’s protocol. An initial PCR step was performed to amplify the CRISPR RNA cassette and to incorporate unique different-length stacking barcodes to each sample. Multiple reactions with 200–400 ng DNA were run in parallel PCRs to ensure ~1000x crRNA library representation. The Illumina sequencing adapters were then added during eight cycles of PCR. All primer sequences are listed in the Supplementary Data 5. The final library product was quantified and qualified using the Bioanalyzer Agilent DNA 1000 kit (Agilent #5067-1504) and Qubit dsDNA High Sensitivity Kit (Invitrogen). Individual samples were pooled in equal molar ratio. The single-gene knockout screening libraries were pair-end sequenced on the MiSeq (Illumina) with MiSeq Reagent V3 150-cycle kit (Illumina), and the double knockout screening libraries were single-end sequenced on the NextSeq (Illumina) with NextSeq Mid-Output V2.5 150-cycle kit (Illumina).

### Single-gene level knockout screening data analysis

Briefly, the sequence data were demultiplexed and trimmed to contain only the CRISPR RNA (either AsCas12a crRNA or SpCas9 sgRNA) sequence cassettes. The read count of each individual CRISPR RNA was calculated with no mismatches permitted in comparison to the reference CRISPR RNA sequences. Individual CRISPR RNAs with a read count lower than 50 in the initial time point were discarded. All samples were normalized to the same number of total reads. A protein domain CS was calculated by averaging the LFC of all CRISPR RNA targeting a given protein domain. LFC = (final CRISPR RNA abundance + 1)/(initial CRISPR RNA abundance). The fold-change of a given CRISPR RNA was capped at a maximum of 100. This data analysis was performed similarly as described previously^18^.

### Double knockout screening data analysis

A BLAST reference index of all sequence combinations was assembled using makeblastdb (version 2.6.0). For each sample, adapter sequences were trimmed using CutAdapt (version 1.16), and then mapped to the BLAST index above using blastn. Blast queried sequence mapping was restricted with three rules: (1) having only one high-scoring dual-crRNA, (2) reporting only one aligned target (“-max_hsps 1 -max_target_seqs 1”), and (3) allowing no more than two mismatching nucleotides (including mismatches, indels and unaligned tails). Read counts across two replicates were condensed for analysis. Dual-crRNA read counts were normalized to one million reads per library. A protein domain CS was calculated by averaging the LFC of all CRISPR RNA targeting a given protein domain. LFC = (final CRISPR RNA abundance + 1)/(initial CRISPR RNA abundance). The fold-change of a given CRISPR RNA was capped at a maximum of 100.

The designed library contained both orientations of each dual-crRNA pair with an experimental crRNA in a fixed position joined to all 22 possible negative control crRNAs in the other position (n.b. exp1_neg1 is not grouped with neg1_exp1 in downstream analysis). Any experimental crRNA with less than four different negative controls in the other position detected on sequencing was eliminated from further analysis (e.g. if only three different negative control crRNAs in position two are detected paired with experimental crRNA “1” in the first position, then all experimental crRNA “1” pairings in either position are eliminated from the study). From the dual-crRNAs that met these criteria, any dual-crRNAs that had less than 8 detected dual-crRNA pairs were further eliminated. In the end, each gene in our analysis had at least two valid crRNAs designed for it.

To quantify the effect of each experimental crRNA, a Gaussian distribution was fitted using all the observed LFC values of the experimental crRNA in combination with all negative control crRNAs. The fitting was performed in a position sensitive way (i.e. ƒ_g_ (exp1_neg) is not the same as ƒ_g_ (neg_exp1)). To estimate the expected phenotypic effect of an experimental–experimental dual-crRNA pairing, the Gaussian distributions of each experimental crRNA paired with negative control crRNAs were multiplied. (i.e. ƒ_g_ (exp1_exp2) = ƒ_g_ (exp1_neg) × ƒ_g_ (neg_exp2) and ƒ_g_ (exp2_exp1) = ƒ_g_ (exp2_neg) × ƒ_g_ (neg_exp1)).

To estimate the expected phenotypic effect at the gene level, a Gaussian distribution model was built based on the combination of all the experimental crRNAs targeting a given gene and the negative control crRNAs. Similar to the analysis at the crRNA level, to estimate the expected phenotypic effect of two experimental genes, two Gaussian distributions of each gene were multiplied. To calculate the observed phenotypic effect of two genes, a Gaussian distribution was fitted by all the CSs of crRNA combinations targeting the two experimental genes. To identify potential synthetic sick/lethal genetic interactions, a Kolmogorov–Smirnov test was performed on the expected Gaussian distribution and the observed Gaussian distribution of two genes paired with an FDR cutoff of 0.05 and a LFC of 2.5.

### Double knockout uncoupling data analysis

A BLAST reference index of all library dual-crRNA cassette sequences and all possible cassette recombination products was assembled using makeblastdb (version 2.6.0). For each sample, adapter sequences were trimmed using CutAdapt (version 1.16), reads shorter than 60 bp were discarded (empty crRNA vector), and filtered reads were mapped to the BLAST index above using blastn. Blast queried sequence mapping was restricted with three rules: (1) having only one high-scoring dual-crRNA, (2) reporting only one aligned target (“-max_hsps 1 -max_target_seqs 1”), and (3) allowing no more than two mismatching nucleotides (including mismatches, indels and unaligned tails). For each sample, the number of reads mapped to each reference sequence was counted. Uncoupling frequency was calculated as the fraction of all reads mapped to a cassette recombination product divided by total reads mapped to any reference sequence.

### Small molecule inhibitor treatment

*Jmjd6*-deficient cells were generated by infecting RN2c12 cells with a validated *Jmjd6* crRNA. crRNA-positive (GFP positivity) cells were selected with G418 to achieve ≥95% purity and validated with TIDE analysis. A control cell line was also generated by transducing a Rosa26 crRNA in the same way. To test the cell growth of crJmjd6- and crRosa26-containing RN2c12 cells upon dBRD9 (provided by Jun Qi) or WM-1119 (Tocris, #6692) treatment, 1000 cells were plated into 48-well plates. Serially diluted concentrations of dBRD9, WM-1119, or 0.1% DMSO (negative control) was used for the treatment. After a 5-day incubation, cell viability was measured using CellTiter-Glo Luminescent Cell Viability Assay kit (Promega #G7570) with Synergy HTX microplate reader (Biotek). A 1:3 ratio of reagent to PBS was used, and all other steps were conducted according to the manufacturer’s protocol.

### RNA-seq and data analysis

RN2c12 cells were harvested on day 6 post infection with indicated crRNAs. To enrich the crRNA-positive population, G418 selection was applied on day 2 post infection. RNA was isolated from crRNA-transduced RN2c12 cells just before the onset of the growth arrest phenotype in order to capture the immediate transcriptional program changes. Total RNA was isolated using Direct-zol^TM^ RNA Miniprep Plus kit (Zymo Research #R2072) following the manufacturer’s protocol. The quality of isolated RNA was verified using the RNA 6000 Nano Bioanalyzer kit (Agilent #5067-1511). Only RNA with a RIN ≥ 9 was used for subsequent library construction. RNA-seq libraries were prepared with the QuantSeq 3′ mRNA-Seq Library Prep Kit FWD for Illumina (Lexogen). 500 ng of total RNA was used for the initial input, and 12 cycles of PCR were used in barcode amplification. Quality of the RNA-seq libraries was assessed using the High Sensitivity DNA Bioanalyzer kit (Agilent # 5067-4626). Libraries were pooled together and sequenced on the NextSeq 500/550 Platform with single-end reads of 75 bases (Illumina).

Sequencing reads were mapped to the reference mouse genome (mm10) using Lexogen Quantseq 2.3.1 FWD platform. Low quality reads, poly (A) read-through, and adapter contamination were removed with BBDuk, and raw reads were mapped to mm10 using STAR Aligner with modified ENCODE settings. HTSeq-count was used to generate gene read count files. Mapped raw reads were subjected to DESeq2 (1.14.1) to identify differentially expressed genes with default settings, and read counts >2 were considered expressed. Genes with |log2FC| ≥ 1 and *p* < 0.05 were considered significantly and strongly up- or downregulated genes. To generate heatmaps, raw read counts were first converted to read per kilobase of transcript per million (RPKM) using the rpkm function in R (version 3.3.2). RPKM ≥ 0.2 were considered expressed. The heatmap was generated using the heatmap.2 function in R, with RPKM in each condition averaged. Gene lists for the Leukemia Stem Cell and Myeloid Differentiation gene set enrichment analyses are provided (Supplementary Data 6).

### Reporting summary

Further information on research design is available in the Nature Research Reporting Summary linked to this article.

## Supplementary information

Supplementary Information

Supplementary Data 1

Supplementary Data 2

Supplementary Data 3

Supplementary Data 4

Supplementary Data 5

Supplementary Data 6

Reporting Summary

Peer Review File

Description of Additional Supplementary Files

## Data Availability

The RNA-seq datasets generated and analyzed during the current study are publicly available in the GEO repository under the accession number GSE141130. Pfam, UniProt, and NCBI RefSeq databases annotating the mouse genome (GRCm38/mm10, Dec. 2011) were queried using the UCSC genome browser (https://genome.ucsc.edu/cgi-bin/hgGateway). All other data generated or analyzed during this study are included in this published article, its Supplementary Information files, and the Source Data file (as indicated in the article). Additional information pertaining to the current study is available from the corresponding author on reasonable request. Source Data are provided with this paper.

## Source data

Source Data

## Code availability

The double knockout screening data analysis script pipeline is available at: [https://github.com/yeminlan/dkoscreen]. The license for this custom software is supplied in the above GitHub repository; provides permissions for modification, distribution, private use, and commercial use all conditioned on license and copyright notice; and limits liability and warranties. Source Data are provided with this paper.
