# Supplementary material for: High-performance CRISPR-Cas12a genome editing for combinatorial genetic screening
Source: Nat Commun. 2020 Jul 13;11:3455. doi: 10.1038/s41467-020-17209-1 (PMC7359328; doi:10.1038/s41467-020-17209-1)
Supplement: Supplementary file 1 — Supplementary Information [file 41467_2020_17209_MOESM1_ESM.pdf]

Supplementary information for:

High-performance CRISPR-Cas12a genome editing for combinatorial genetic screening

Gier & Budinich et al.

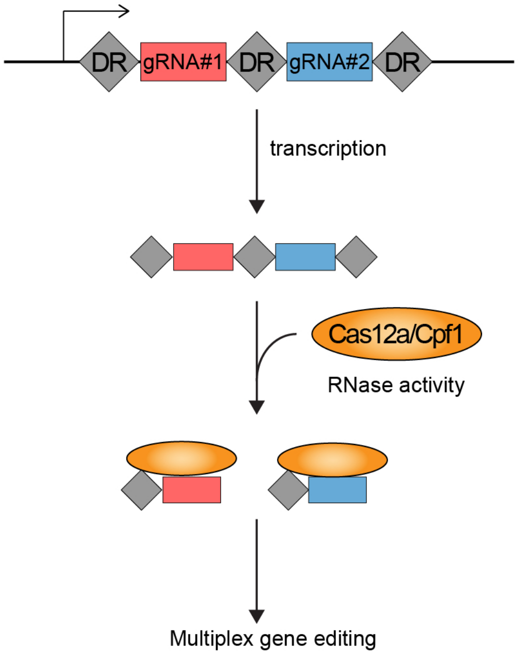

Supplementary Figure 1

Multi-gene editing by CRISPR-Cas12a/Cpf1 system.

Schematic demonstrating how CRISPR-Cas12a processes a single transcript using its intrinsic RNase activity for multi-gene editing. DR signifies the “direct repeat” portion of a targeting CRISPR RNA that mediates binding of Cas12a to the RNA molecule. “gRNA (guide RNA)” signifies the unique portion of a targeting CRISPR RNA that mediates site-specific genomic targeting of the Cas12a nuclease.

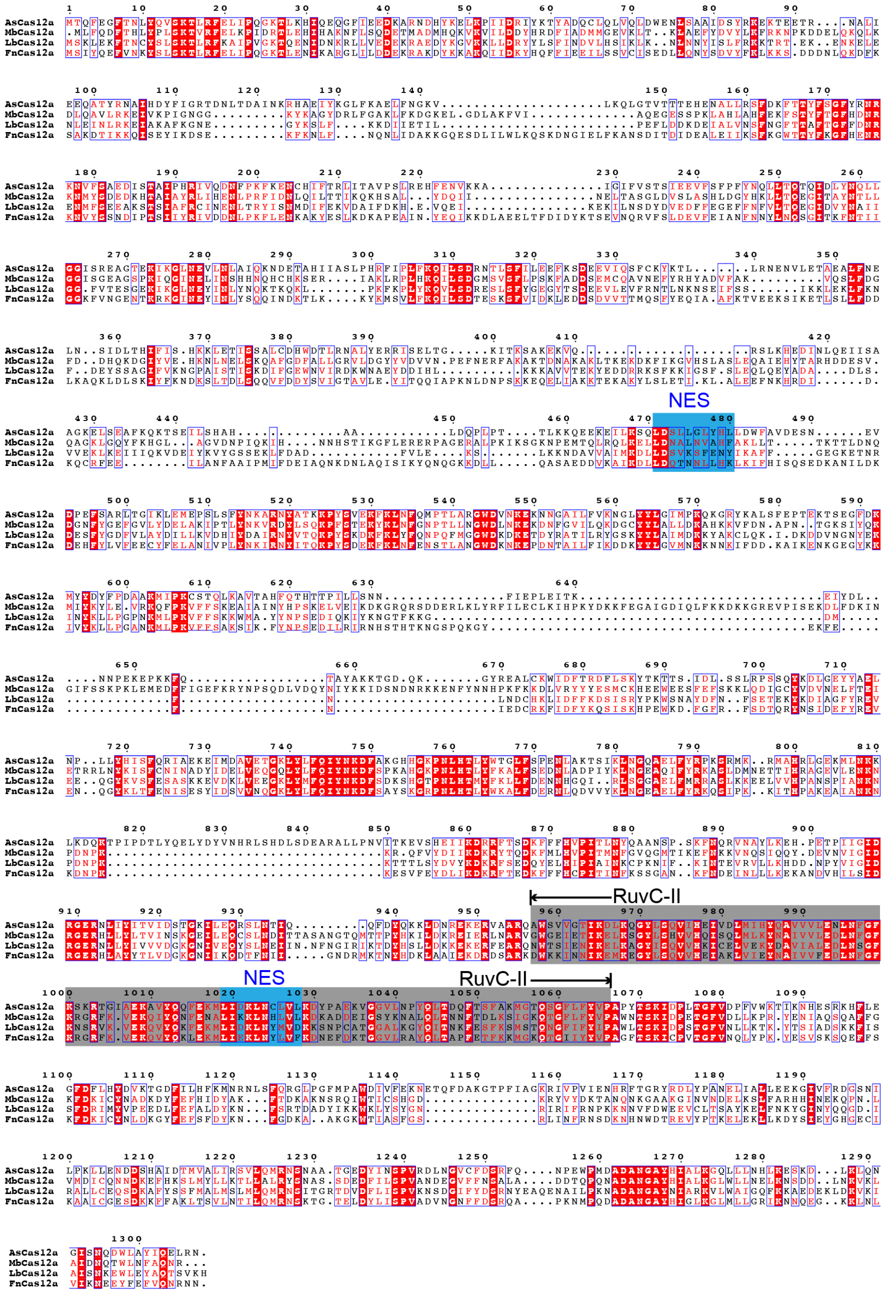

Supplementary Figure 2

AsCas12a contains two potential nuclear export signal (NES) sequences.

Multiple sequence alignment of the amino acid sequences of AsCas12a, MbCas12a, LbCas12a, and FnCas12a. As, *Acidaminococcus sp*; Mb, *Moraxella bovoculi*; Lb, *Lachnospiraceae bacterium*; Fn, *Francisella novicida*. The multiple sequence alignment was performed using Clustal Omega (https://www.ebi.ac.uk/Tools/msa/clustalo/). Conserved residues are labeled with a red background and white font. Residues with chemically similar side chains are indicated with an outline and red font. The conserved catalytic RuvC-II domain is highlighted with a grey box. Predicted NES sequences using the NetNES website tool (http://www.cbs.dtu.dk/)[1] are highlighted in blue. The canonical NES signal is LxxxLxxLxL. While AsCas12a contains 2 potential NES sequences, there are no predicted NES sequences in SpCas9.

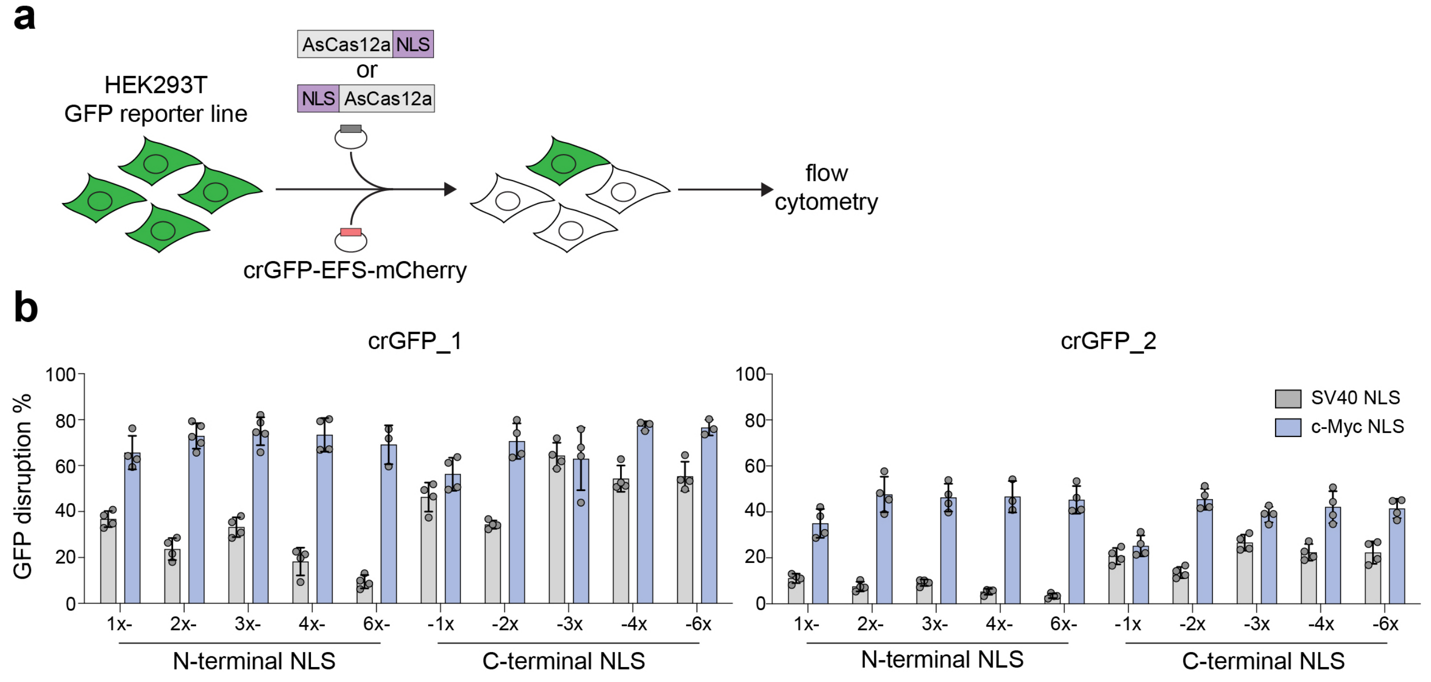

Supplementary Figure 3

NLS sequence, number, and position affect AsCas12a knockout efficiency in mammalian cells.

(**a**) Experimental workflow of GFP disruption assay to evaluate AsCas12a knockout efficiency. GFP-expressing HEK293T cells were transiently transfected with plasmids encoding (1) AsCas12a with NLS variations and (2) GFP-targeting crRNA. Flow cytometry-based tracking of the GFP-positive population over a period of time was used to calculate the GFP disruption phenotype of each indicated AsCas12a-NLS variant. (**b**) Percent GFP disruption as a function of NLS sequence (SV40 NLS vs. c-Myc NLS), number (1-6 NLS repeats), and position (N-terminal or C-terminal to AsCas12a). Data shown for 2 separate GFP-targeting crRNA sequences (crGFP_1 and crGFP_2). *n* = 3-5 independent biological replicate samples. All error bars shown represent mean +/- SD. Source data are provided as a Source Data File.

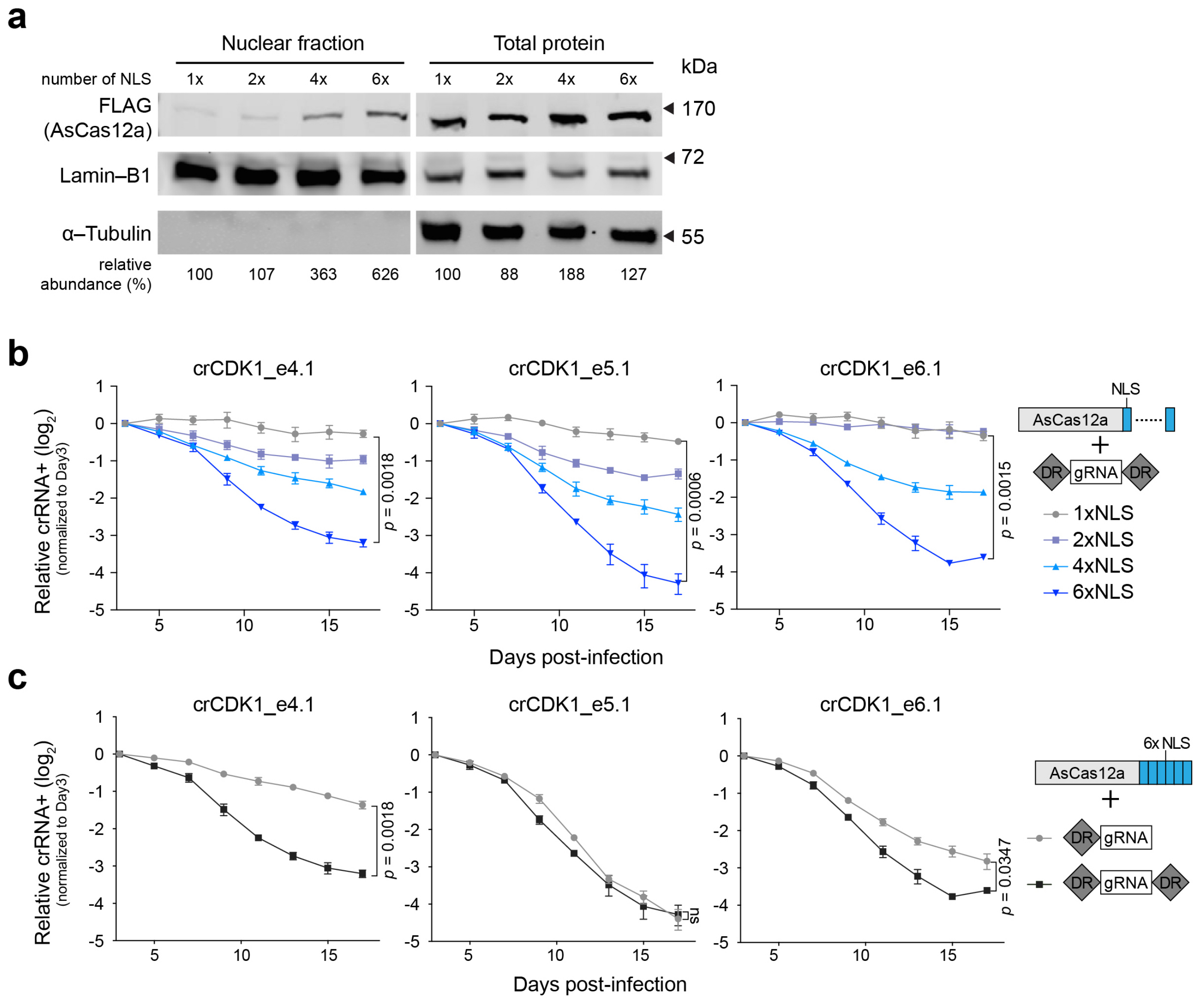

Supplementary Figure 4

Appending NLS and DR sequences improves AsCas12a knockout efficiency in mammalian cells.

(**a**) Western blotting of AsCas12a, Lamin-B1, and α-Tubulin levels in both nuclear fraction and whole-cell lysates prepared from K562c12 cells. K562 cells were transduced with the indicated AsCas12a constructs containing a variable number of NLS repeats. A representative experiment of two independent replicates is shown (n = 2). The relative abundance of AsCas12a (FLAG) protein is quantified using gel densitometry (ImageJ) and normalized first to Lamin-B1 and then to the corresponding 1x NLS samples. (**b**,**c**) Cellular competition assay to characterize the knockout efficiency of the AsCas12a system with a variable number of NLSs (**b**) and with or without an additional DR 3' of the crRNA (**c**). Plotted are the crRNA+ cell populations (normalized to the day 3 measurement) at the indicated timepoints. Three crRNAs were designed targeting the kinase domain of *CDK1*, an essential gene for cell cycle regulation. 'e' represents the exon. n = 3 independent biological replicate samples. ns = not significant; p-values are indicated (two-tailed Welch's unpaired t-test). Error bars smaller than symbol width not shown. All error bars shown represent mean +/- SD. Source data are provided as a Source Data File.

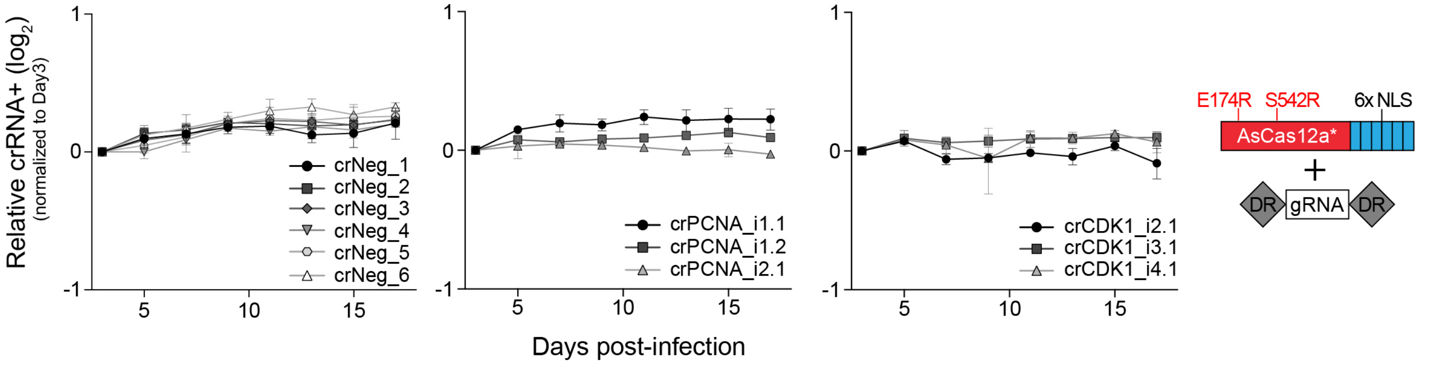

Supplementary Figure 5

Knockout by opAsCas12a with negative control crRNAs does not significantly affect cellular proliferation in K562 cells.

Cellular competition assay of K562c12 cells transduced with indicated crRNAs. Plotted are the crRNA+ cell populations (normalized to the day 3 measurement) at indicated timepoints. crNeg1-6 are non-targeting crRNAs. Additional crRNAs were designed to target intronic regions of the essential genes *PCNA* and *CDK1*. 'i' represents the intron. n = 3 independent biological replicate samples. Error bars smaller than symbol width not shown. All error bars shown represent mean +/- SD. Source data are provided as a Source Data File.

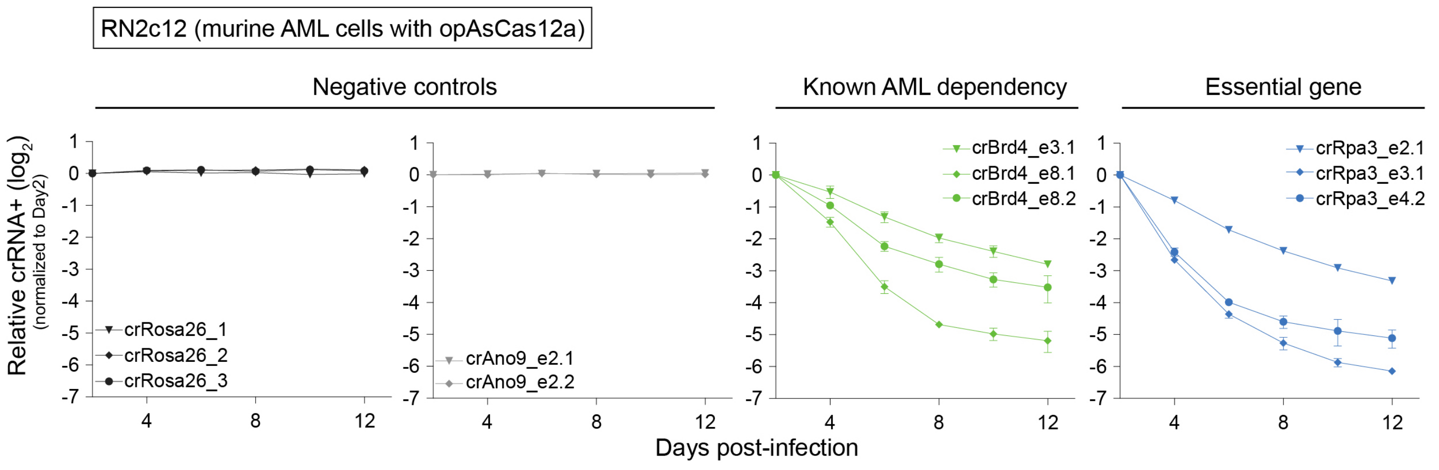

Supplementary Figure 6

opAsCas12a shows robust knockout efficiency in RN2 cells.

Cellular competition assay of RN2c12 cells transduced with indicated crRNAs. Plotted are the crRNA+ cell populations (normalized to the day 2 measurement) at indicated timepoints. crRNAs targeting the *Rosa26* and *Ano9* loci serve as negative controls. crRNAs targeting *Brd4*, a known Mll-Af9 leukemia dependency, and *Rpa3,* an essential gene for cell replication, serve as positive controls. ‘e’ represents the exon. n = 3 independent biological replicate samples. Error bars smaller than symbol width not shown. All error bars shown represent mean +/- SD. Source data are provided as a Source Data File.

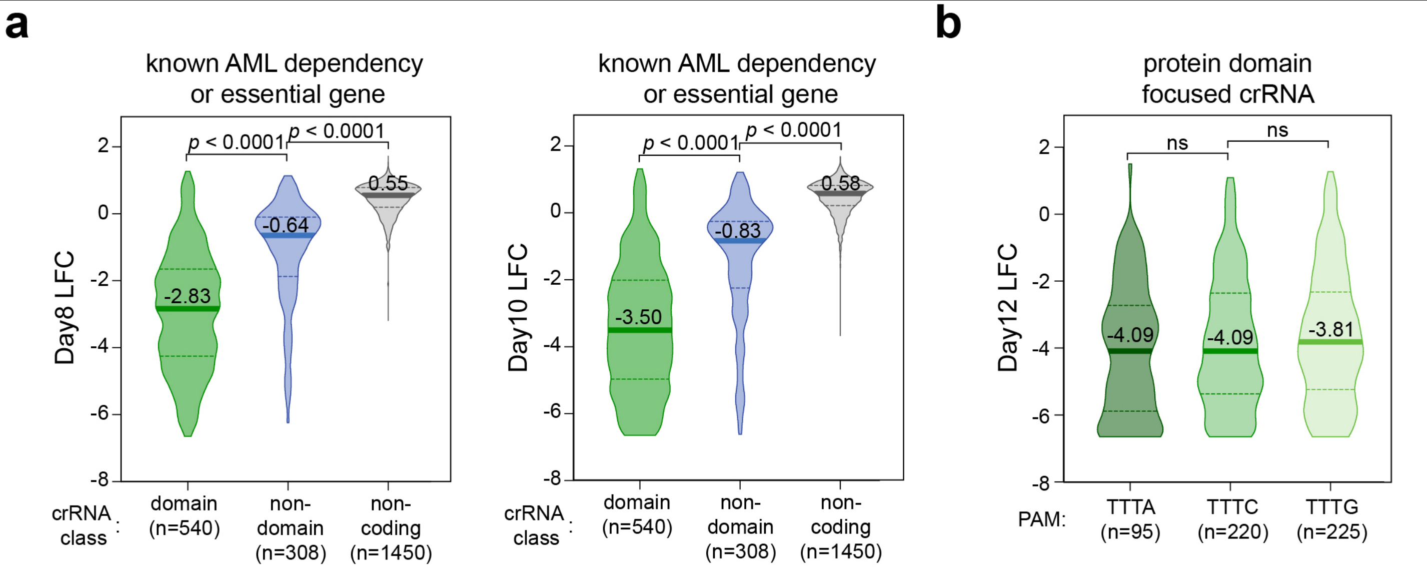

Supplementary Figure 7

crRNA targeting conserved protein domains increase functional gene knockout efficiency in pooled dropout genetic screens.

Comparative functional knockout efficiency of known AML dependency and essential target genes with variable screen duration, crRNA targeting position, or crRNA PAM. Violin plot demonstrates median (thick line, value listed), interquartile (dashed lines), and distribution of guide-wise $\log_2$ fold-change of two replicate screens. (**a**) Log_2 fold-change of crRNAs targeting structural protein domains, non-domain coding regions, and non-coding regions at 8 and 10 days post-infection. (**b**) Log_2 fold-change of protein domain-targeting crRNAs with either a TTTA, TTTC, or TTTG PAM sequence. *p*-values are indicated and ns = not significant (two-tailed Mann-Whitney U-test). Source data are provided as a Source Data File.

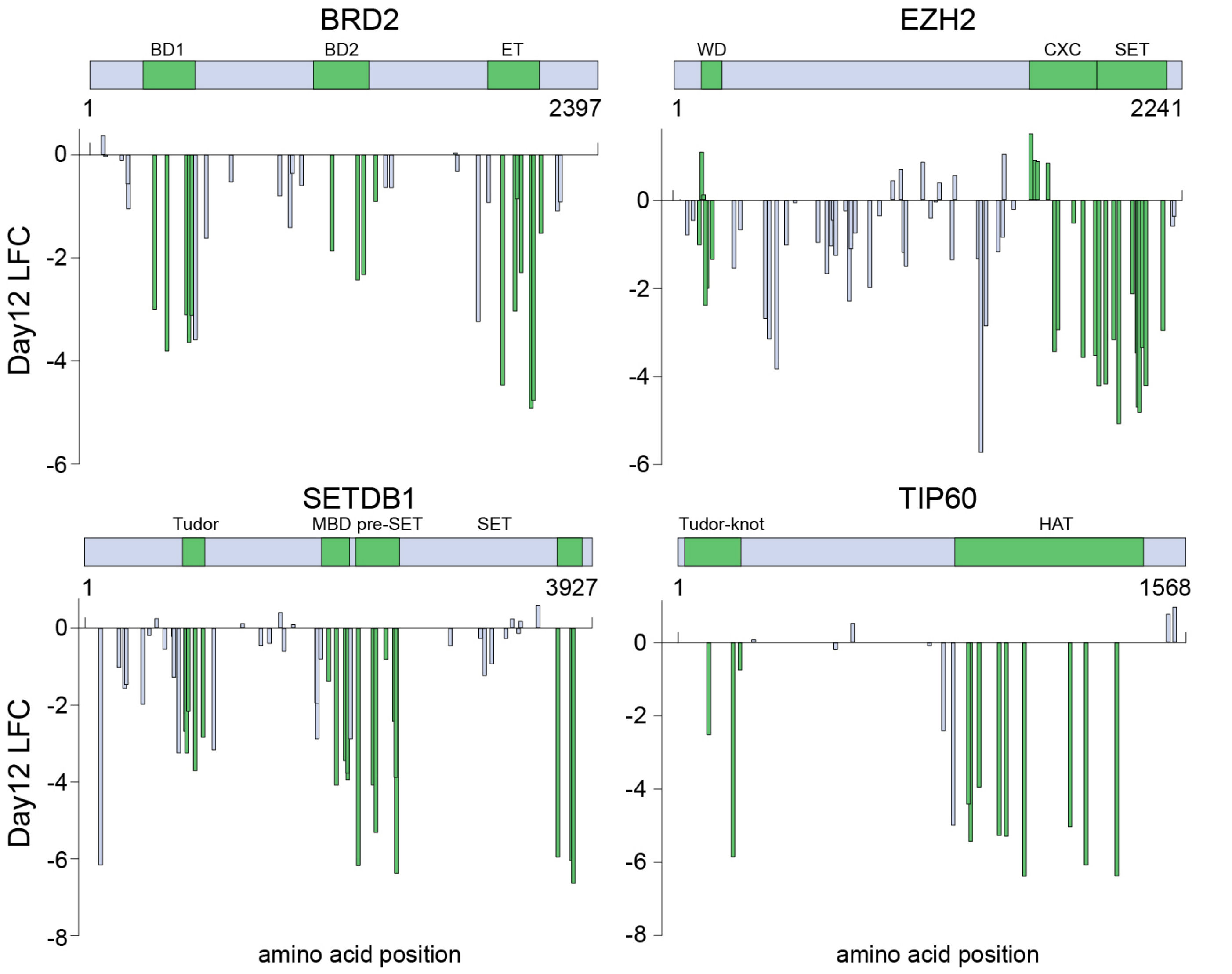

Supplementary Figure 8

Individual examples of functional protein domain targeting crRNAs lead to a higher proportion of null mutations and an enhanced severity of dropout.

The location of each crRNA cleavage site within the BRD2, EZH2, SETDB1, and TIP60 coding sequences is indicated along the x-axis. Plotted is the mean $\log_2$ fold-change value of two biological replicates. n = 2 independent samples. crRNAs targeting inside or outside of conserved protein domains are represented by green or blue bars, respectively. Source data are provided as a Source Data File.

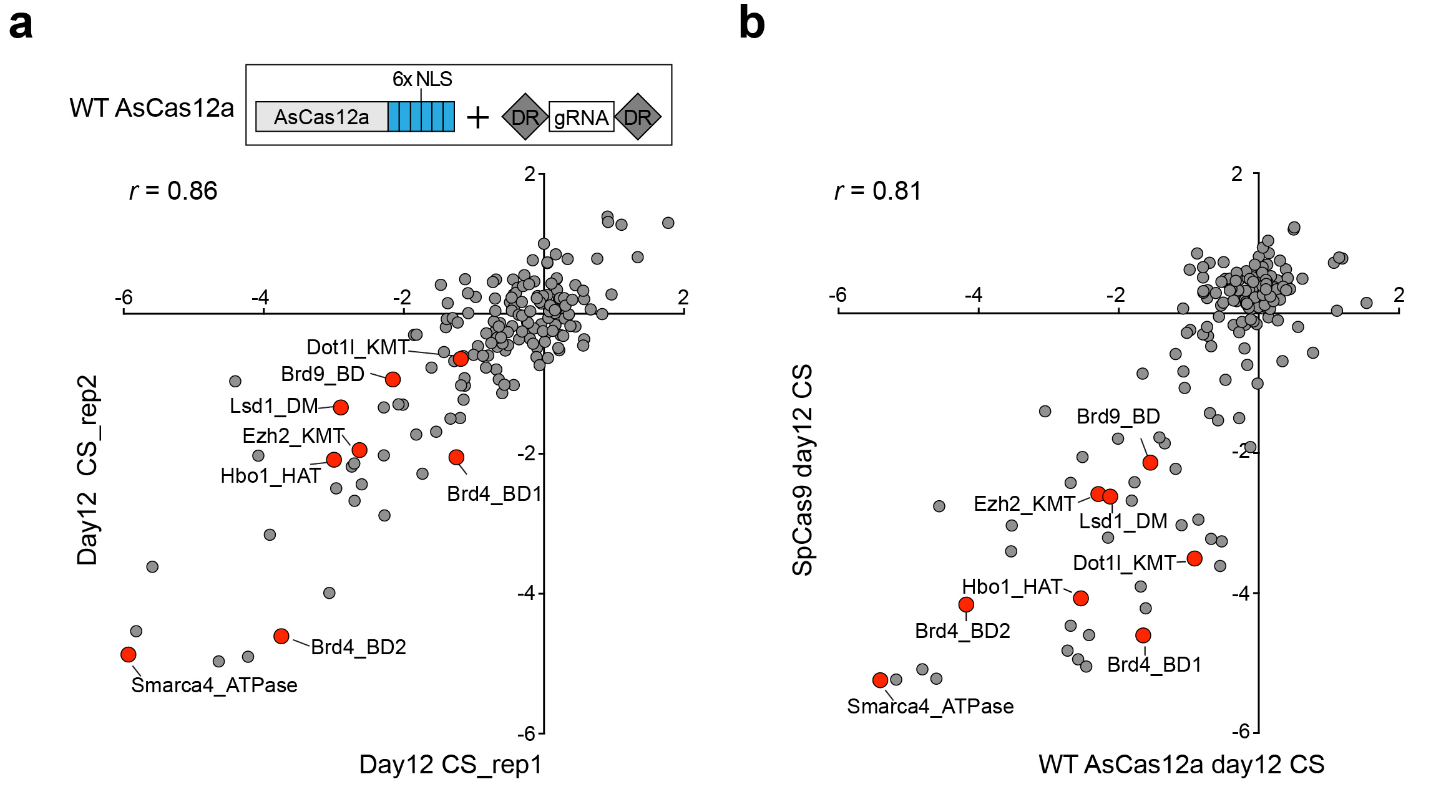

Supplementary Figure 9

Evaluation of the performance of WT AsCas12a in dropout screens to identify epigenetic dependencies in RN2 cells.

A customized AsCas12a crRNA library against 155 functional protein domains involved in epigenetic regulation was constructed. The library contains 3-5 crRNAs per domain. crRNAs were designed to target functional protein domains, yielding 787 crRNAs total including positive and negative controls. RN2 cells were transduced with pooled AsCas12a crRNA or SpCas9 sgRNA libraries targeting epigenetic regulatory domains at low multiplicity of infection to ensure single copy viral integration. (**a**-**b**). (**a**) Scatter plot compares the protein domain CSs of two independent replicate screens using WT AsCas12a. (**b**) Comparison of WT AsCas12a and SpCas9 in pooled dropout screens against epigenetic regulators. Scatter plot compares the CSs from screens in RN2 cells. Plotted are the averages of two independent biological replicate samples. (**a**-**b**) Red dots label known cancer drug targets in this Mll-Af9 leukemia model. Pearson correlation coefficients (*r*) shown. Source data are provided as a Source Data File.

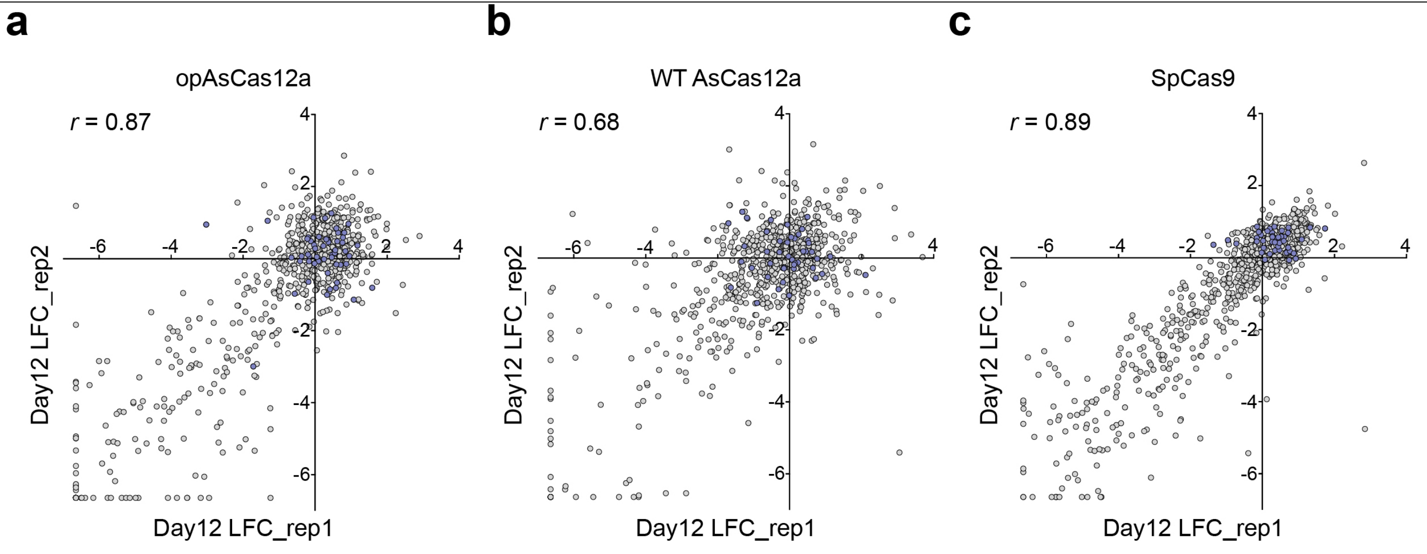

Supplementary Figure 10

CRISPR RNA-wise comparison of opAsCas12, WT AsCas12a, and SpCas9 in pooled dropout screens against epigenetic regulators.

CRISPR RNA-wise results of opAsCas12a, WT AsCas12, and SpCas9 pooled dropout screens to identify epigenetic dependencies in RN2 cells. (**a**-**b**) A customized AsCas12a crRNA library against 155 functional protein domains involved in epigenetic regulation was constructed. The library contains 3-5 crRNAs per domain. crRNAs were designed to target functional protein domains, yielding 787 crRNAs total including positive and negative controls. (**a**-**c**) RN2 cells were transduced with pooled AsCas12a crRNA or SpCas9 sgRNA libraries targeting epigenetic regulatory domains at low multiplicity of infection to ensure single copy viral integration. Scatter plots compare the LFCs of two independent biological replicate samples. Pearson correlation coefficients (*r*) shown. The LFC of a given CRISPR RNA is capped at a maximum of 100. The blue dots label negative control CRISPR RNAs. Source data are provided as a Source Data File.

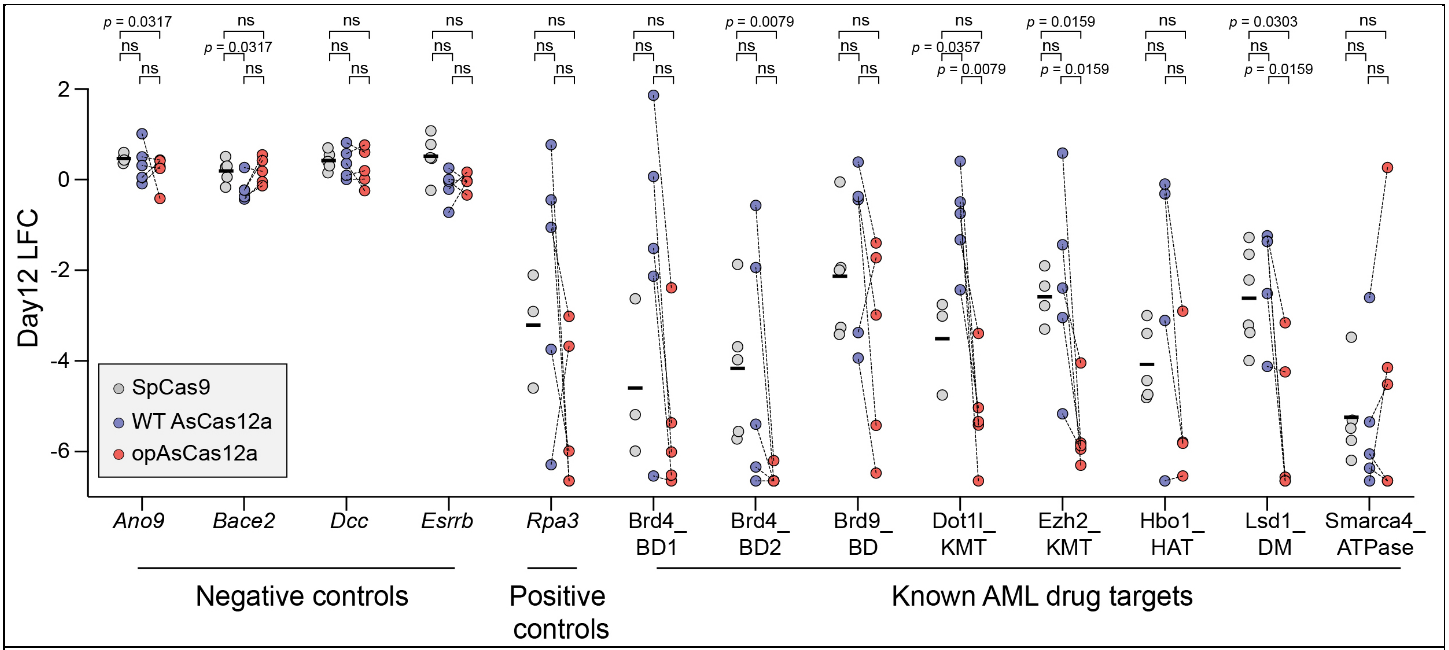

Supplementary Figure 11

opAsCas12a performs as well as SpCas9 in a pooled library dropout screen against epigenetic regulators.

Log_2 fold-change of cells containing specific CRISPR RNAs from opAsCas12a-, WT AsCas12a- and SpCas9-based dropout screens in RN2 cells. The LFC of a given CRISPR RNA is capped at a maximum of 100. Bold lines represent the CS targeting the indicated gene or protein domain. The dotted line links identical crRNA between the WT AsCas12a and opAsCas12a screens. Plotted is the mean LFC value of two independent biological replicate samples. *p*-values are indicated and ns = not significant (two-tailed Mann-Whitney U-test). Source data are provided as a Source Data File.

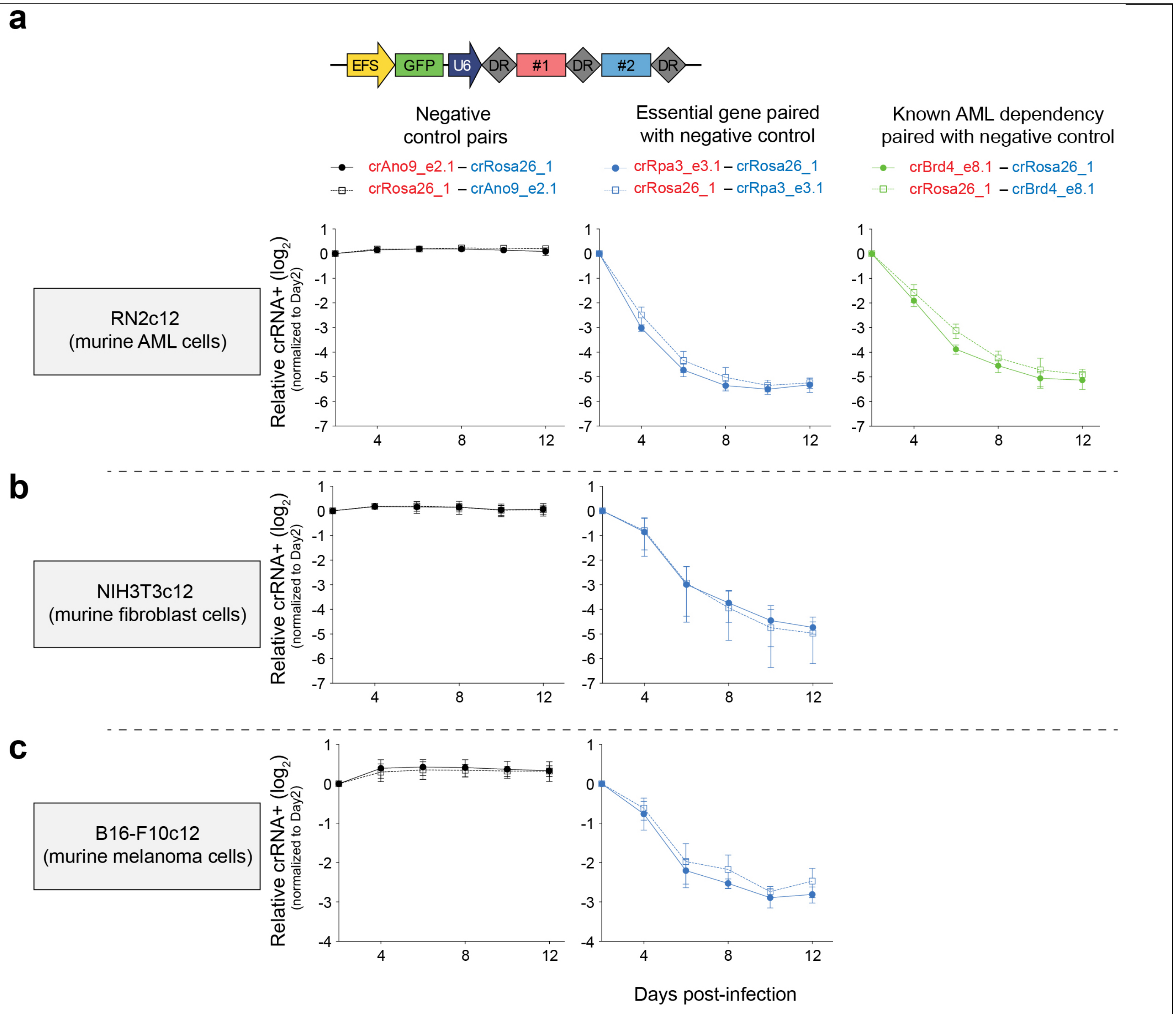

Supplementary Figure 12

opAsCas12a dual-crRNA system performs efficiently across murine cell lines.

Targeting essential genes shows robust knockout activity across RN2c12, NIH3T3c12, and B16-F10c12 cell lines. A decreased cellular proliferation phenotype was validated in leukemia cells for leukemia-specific genetic dependencies via decreased proliferation of RN2c12 upon knockout of the known AML dependency *Brd4*. (**a**) RN2c12 cells were lentivirally transduced with indicated dual-guides. Gene targets were paired with non-targeting crRNA for the Rosa26 locus. Plotted are dual-crRNA (i.e. Double) positive cells normalized to the day 2 measurement at the indicated timepoints. Shown is the average of three independent biological replicate samples for each guide pairing. (**b-c**) NIH3T3c12 or B16-F10c12 cells were lentivirally transduced with indicated dual-guides. Gene targets were paired with non-targeting crRNA for the Rosa26 locus. Shown is the average of three independent biological replicate samples for each guide pairing. Error bars smaller than symbol width not shown. All error bars shown represent mean +/- SD. Source data are provided as a Source Data File.

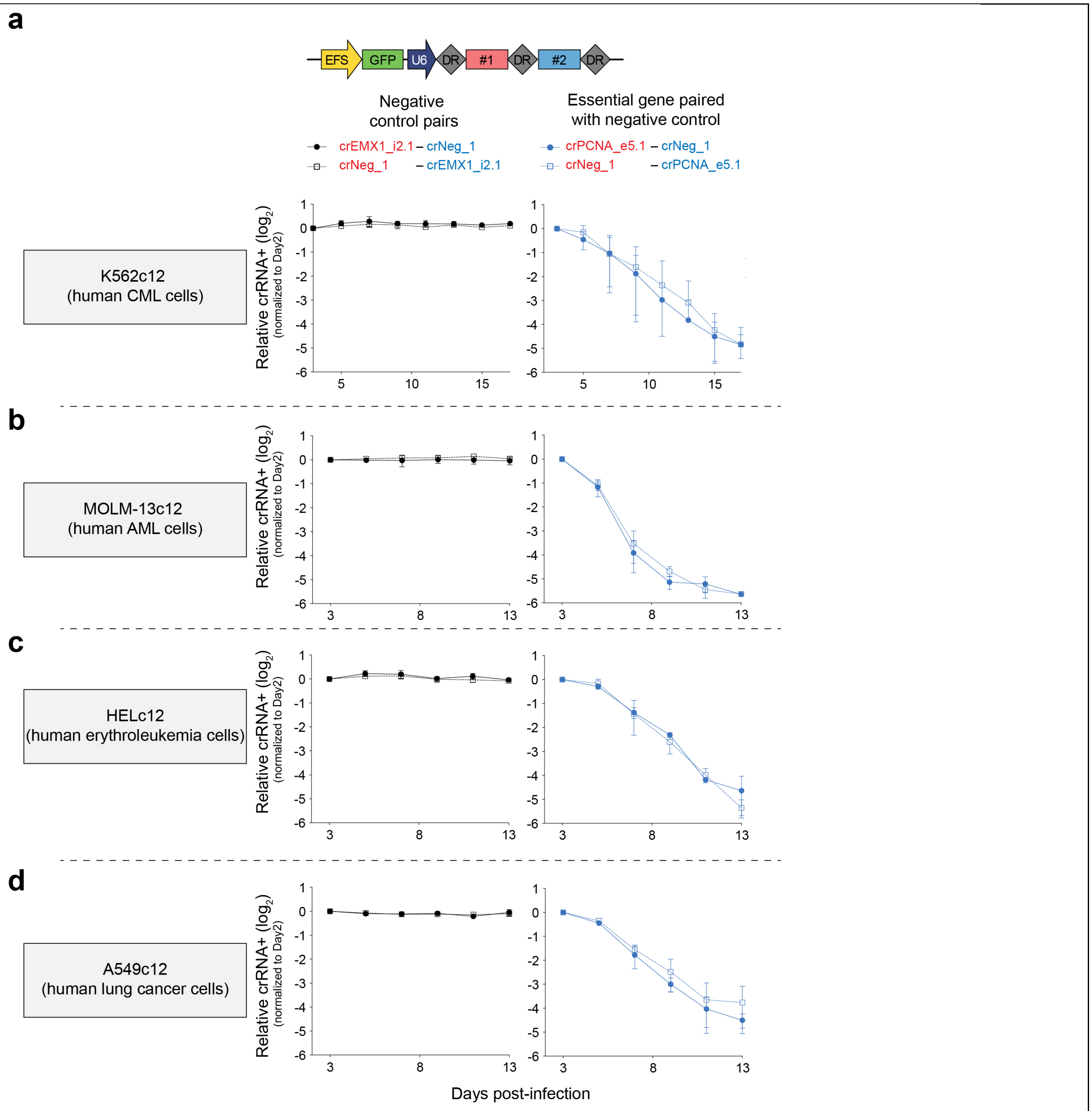

Supplementary Figure 13

opAsCas12a dual-crRNA system performs efficiently across human cell lines.

Targeting essential genes shows robust knockout activity across K562c12, MOLM-13c12, HELc12, and A549c12 cell lines. (**a**) K562c12, (**b**) MOLM-13c12, (**c**) HELc12, and (**d**) A549c12 cells were lentivirally transduced with indicated dual-crRNAs. Gene targets were paired with non-targeting crRNA for the Rosa26 locus. Plotted are dual-crRNA (i.e. Double) positive cells normalized to the day 2 measurement at the indicated timepoints. Shown is the average of three independent biological replicate samples for each guide pairing. Error bars smaller than symbol width not shown. All error bars shown represent mean +/- SD. Source data are provided as a Source Data File.

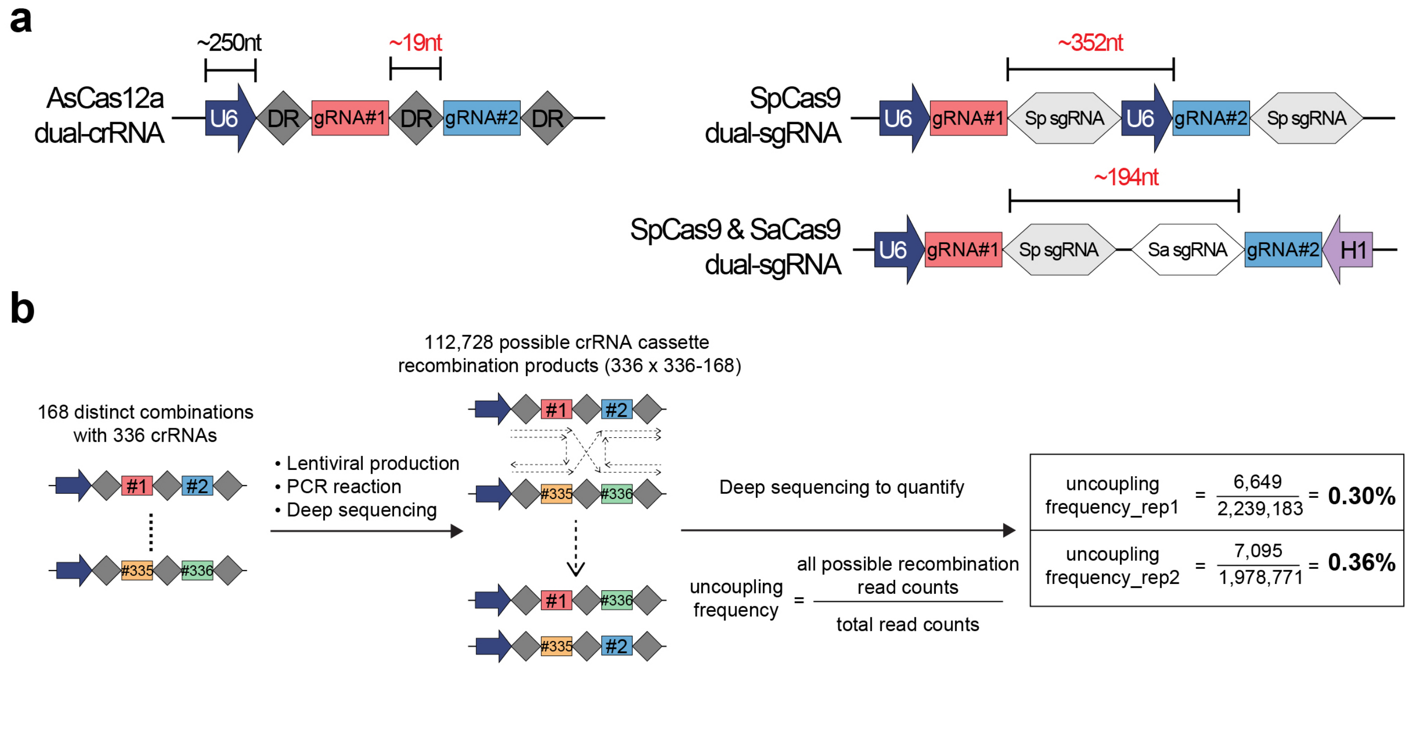

Supplementary Figure 14

opAsCas12a allows for dual-crRNA dropout screening with low guide uncoupling due to crRNA cassette recombination.

(**a**) Schematic comparison of CRISPR RNA transcript expression vector architecture for opAsCas12a- and SpCas9-based double gene knockout. The opAsCas12a system requires a short DR sequence of 19-20 nucleotides (nt) for double gene knockout in comparison to the ~194-352nt of Cas9-based systems. (**b**) Evaluation of the recombination frequency of dual-crRNAs in opAsCas12a system. Experimental workflow to quantify the uncoupling frequency of dual-crRNAs by deep sequencing. Uncoupling frequency is defined as the number of incorrect read counts within the total number of read counts. The uncoupling frequency is shown in a 336-crRNA library.

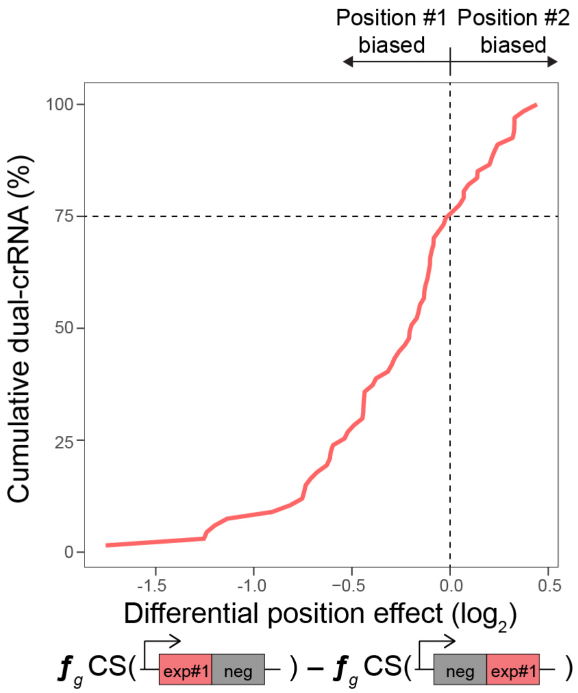

Supplementary Figure 15

Position effect in the enAsCas12a dual-crRNA expression vector in pooled screening.

Cumulative curve that describes the percentage of dual-crRNAs with a preferential proliferation effect from a crRNA in either the first or second position of a dual-crRNA array. Positional difference on the x-axis is computed by subtracting the effect of a crRNA when paired with negative controls in the first position vs. when paired with negative controls in the second position. Negative values indicate a crRNA with a proliferation bias in the first position and positive values indicate a guide bias in the second position. The y-axis shows the percentage of dual-crRNAs less than or equal to the indicated positional difference.

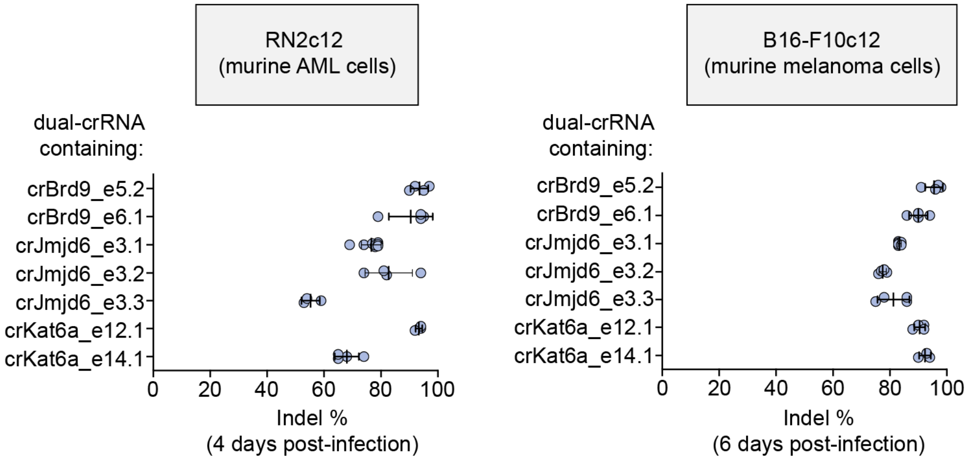

Supplementary Figure 16

Tracking of Indels by DEcomposition (TIDE)[2] mutagenesis assay of opAsCas12a crRNAs.

Dot plot depicts Inference of CRISPR Edits, ICE, (indel%) score for dual-crRNA. RN2c12 (**a**) or B16-F10c12 (**b**) cells were lentivirally transduced with 4 independent dual-crRNA used in **Fig. 3c**, all containing the indicated crRNA in either the first or second position. Genomic DNA was isolated from RN2c12 on day 4 and from B16-F10c12 on day 6 for target region amplification. Each dot within an indicated crRNA cluster represents a different crRNA pair. The negative selection phenotype of dual-experimental crRNA in RN2c12 cells requires early timepoint genomic DNA isolation, and therefore likely underestimates the gene editing efficiency in RN2c12 cells. ICE scores are calculated via Sanger sequencing and analyzed using Synthego online ICE analysis tool. All error bars shown represent mean +/- SD. Source data are provided as a Source Data File.

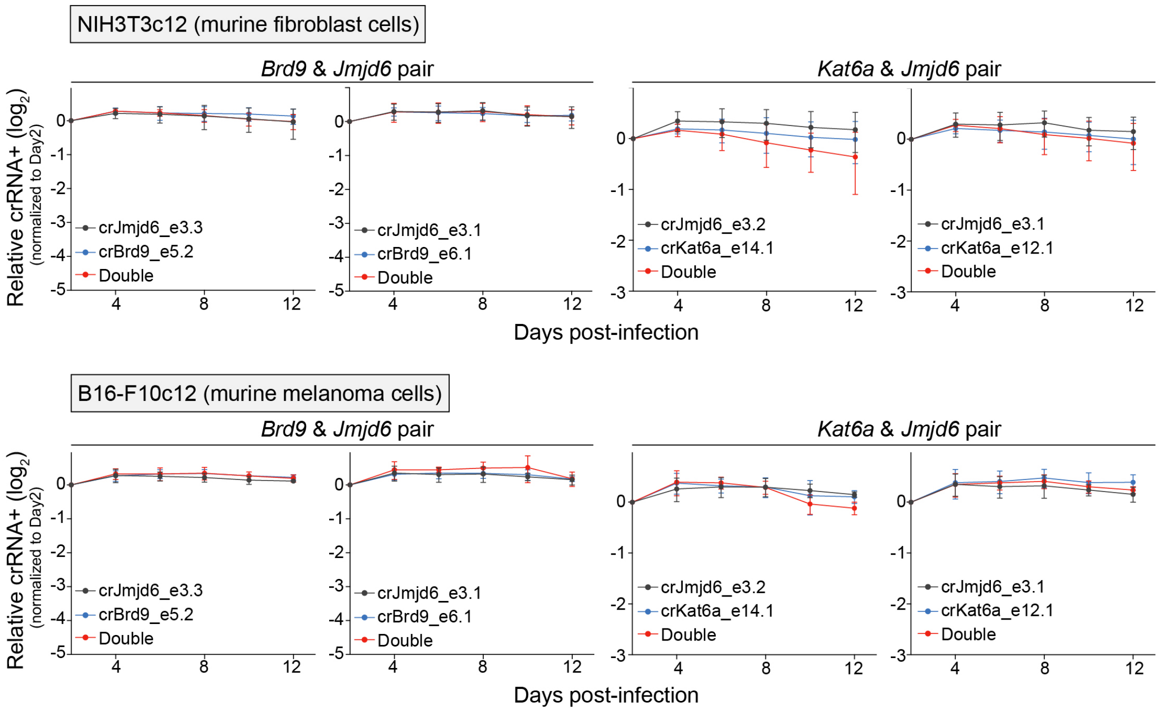

Supplementary Figure 17

Validation of potential synergistic leukemia-specific genetic interactions across cell lines.

Cellular competition assays in (**a**) NIH3T3c12 fibroblasts and (**b**) B16-F10c12 melanoma cells to validate potential *Brd9&Jmjd6* and *Kat6a&Jmjd6* genetic interactions identified by the combinatorial screen. Plotted are single- (e.g. crJmjd6_e3.3) or dual-crRNA (e.g. Double) positive cells normalized to the day 2 measurement at the indicated timepoints. The second crRNA in the dual-crRNA cassette for single-crRNA samples targets the negative control Rosa26 locus. For each of 3 independent biological replicate samples, values from both orientation permutations of the dual-crRNA cassette are averaged to correct for any crRNA position effect. Position-corrected data from the 3 biological replicates are plotted as averages. Error bars smaller than symbol width not shown. All error bars shown represent mean +/- SD. Source data are provided as a Source Data File.

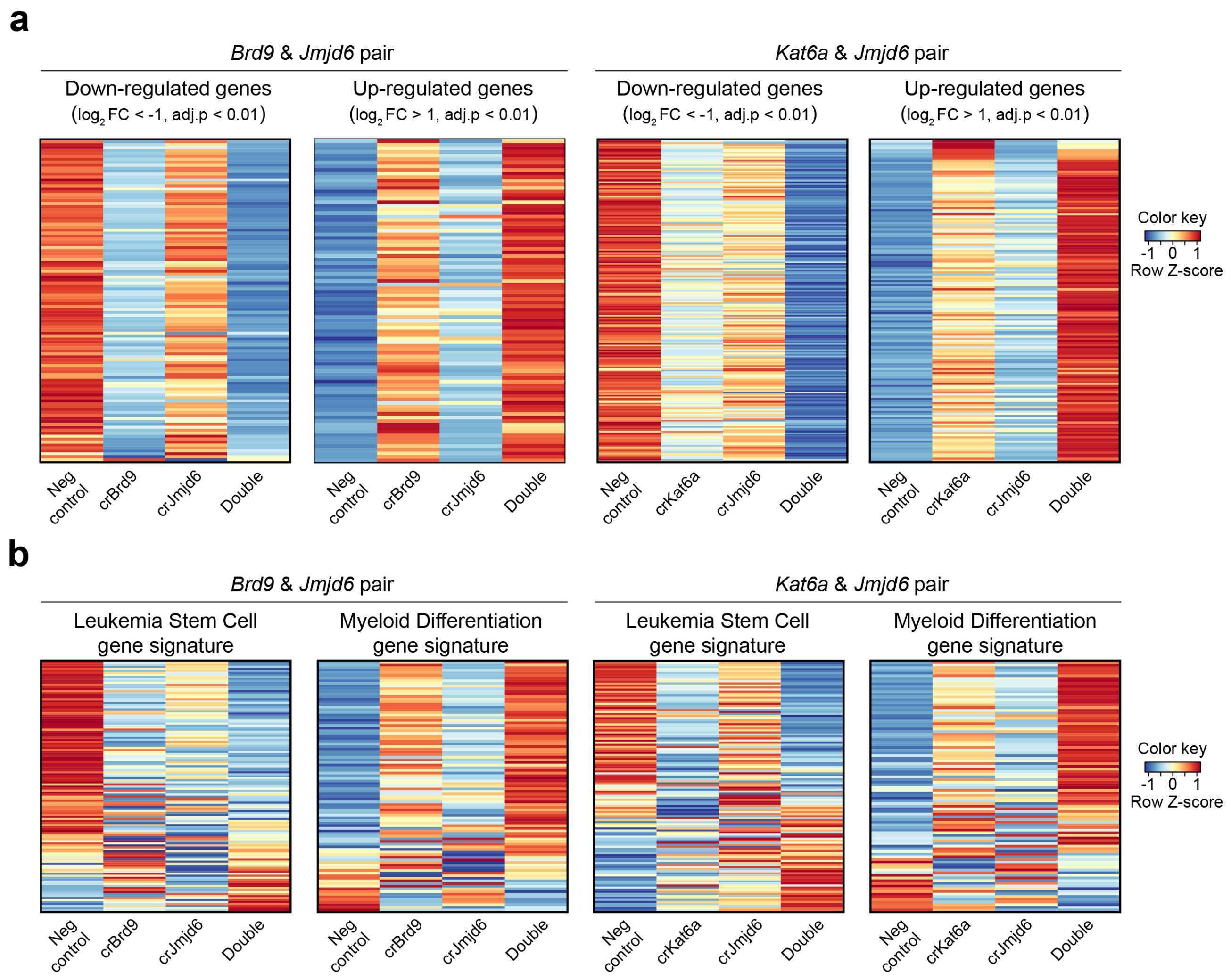

Supplementary Figure 18

RNA-seq analysis of *Brd9&Jmjd*6 and *Kat6a&Jmjd6* synthetic sick/lethal interactions in RN2c12 cells.

RNA-seq heatmap of gene expression changes following transduction with indicated dual-crRNAs in RN2c12 cells. The second crRNA in the dual-crRNA cassette for single-crRNA samples targets the negative control Rosa26 locus. RNA was isolated from RN2c12 cells 6 days post-transduction with indicated dual-crRNAs. Results are the average of 2-6 independent dual-crRNAs. (**a**) RNA-seq data depicting the more than 2-fold significantly up-regulated and down-regulated genes in double knockout with indicated dual-crRNAs in RN2c12 cells as depicted in **Fig. 3e**. (**b**) Genes related to leukemia stem cell signature and myeloid differentiation programs are plotted and ranked based on fold change in expression. The order of genes in the heatmaps is consistent among heatmaps from a given gene pair but differs between the *Brd9&Jmjd6* pair and the *Kat6a&Jmjd6* pair.

Supplementary References

1. la Cour, T. et al. Analysis and prediction of leucine-rich nuclear export signals. *Protein engineering, design & selection : PEDS* **17**, 527-536 (2004).
2. Brinkman, E.K., Chen, T., Amendola, M. & van Steensel, B. Easy quantitative assessment of genome editing by sequence trace decomposition. *Nucleic acids research* **42**, e168 (2014).
