# Supplementary material for: High-performance CRISPR-Cas12a genome editing for combinatorial genetic screening
Source: Nat Commun. 2020 Jul 13;11:3455. doi: 10.1038/s41467-020-17209-1 (PMC7359328; doi:10.1038/s41467-020-17209-1)
Supplement: Supplementary file 8 — Reporting Summary [file 41467_2020_17209_MOESM8_ESM.pdf]

nature research

Corresponding author(s): Junwei Shi
Last updated by author(s): Jun 9, 2020

Reporting Summary

Nature Research wishes to improve the reproducibility of the work that we publish. This form provides structure for consistency and transparency in reporting. For further information on Nature Research policies, see our Editorial Policies and the Editorial Policy Checklist.

Statistics

For all statistical analyses, confirm that the following items are present in the figure legend, table legend, main text, or Methods section.

n/a	Confirmed	
☐	☒	The exact sample size (*n*) for each experimental group/condition, given as a discrete number and unit of measurement
☐	☒	A statement on whether measurements were taken from distinct samples or whether the same sample was measured repeatedly
☐	☒	The statistical test(s) used AND whether they are one- or two-sided<br>*Only common tests should be described solely by name; describe more complex techniques in the Methods section.*
☒	☐	A description of all covariates tested
☐	☒	A description of any assumptions or corrections, such as tests of normality and adjustment for multiple comparisons
☐	☒	A full description of the statistical parameters including central tendency (e.g. means) or other basic estimates (e.g. regression coefficient) AND variation (e.g. standard deviation) or associated estimates of uncertainty (e.g. confidence intervals)
☐	☒	For null hypothesis testing, the test statistic (e.g. *F*, *t*, *r*) with confidence intervals, effect sizes, degrees of freedom and *P* value noted<br>*Give P values as exact values whenever suitable.*
☒	☐	For Bayesian analysis, information on the choice of priors and Markov chain Monte Carlo settings
☒	☐	For hierarchical and complex designs, identification of the appropriate level for tests and full reporting of outcomes
☐	☒	Estimates of effect sizes (e.g. Cohen's *d*, Pearson's *r*), indicating how they were calculated

Our web collection on statistics for biologists contains articles on many of the points above.

Software and code

Policy information about availability of computer code

Data collection	General data collection was completed in Microsoft Excel for Mac (version 14.7.7). The Guava Easycyte 10HT instrument runs Guava Express Pro (version 3.3) and Guava InCyte (version 3.3) for data collection. The Licor Odyssey runs Image Studio (version 2.0). The Agilent Bioanalyzer runs 2100 Expert (version B.02.08.SI648). The Biotek Synergy microplate reader runs Gen5 (version 2.04).
Data analysis	General data analysis was completed in Microsoft Excel for Mac (version 14.7.7). As specified in Methods, additional commercially available data analysis software included GraphPad Prism (version 5), ImageJ (version 1.46R), GSL Biotech SnapGene (version 2.3.2), Synthego ICE Analysis (https://ice.synthego.com/#/), Benchling (https://www.benchling.com/), DOE Joint Genome Institute BBDuk (version 35.92), STAR Aligner (version 2.5.2a), DESeq2 (version 1.14.1), and R (version 3.3.2).<br><br>The custom double knockout screening data analysis script pipeline will be available at: [https://github.com/yeminlan/dkoscreen]. This custom software was tested and implemented with R (version 3.3.1 with packages ggplot2_3.3.0, data.table_1.12.8 and plyr_1.8.6), CutAdapt (version 1.16), blast (version 2.6.0), and GNU bash (version 4.2.46(2)-release (x86_64-redhat-linux-gnu)).

For manuscripts utilizing custom algorithms or software that are central to the research but not yet described in published literature, software must be made available to editors and reviewers. We strongly encourage code deposition in a community repository (e.g. GitHub). See the Nature Research guidelines for submitting code & software for further information.

Data

Policy information about availability of data

All manuscripts must include a data availability statement. This statement should provide the following information, where applicable:

- Accession codes, unique identifiers, or web links for publicly available datasets
- A list of figures that have associated raw data
- A description of any restrictions on data availability

The RNA-seq datasets generated and analyzed during the current study are publicly available in the GEO repository under the accession number GSE141130 (https://www.ncbi.nlm.nih.gov/geo/query/acc.cgi?acc=GSE141130). Pfam, UniProt, and NCBI RefSeq databases annotating the mouse genome (GRCm38/mm10, Dec. 2011) were queried using the UCSC genome browser (https://genome.ucsc.edu/cgi-bin/hgGateway). All other data generated or analyzed during this study are included in this published article, its supplementary information files, and the Source Data File (as indicated in the article). Additional information and materials pertaining to the current study are available from the corresponding author on reasonable request.

Field-specific reporting

Please select the one below that is the best fit for your research. If you are not sure, read the appropriate sections before making your selection.

☒ Life sciences ☐ Behavioural & social sciences ☐ Ecological, evolutionary & environmental sciences

For a reference copy of the document with all sections, see nature.com/documents/nr-reporting-summary-flat.pdf

Life sciences study design

All studies must disclose on these points even when the disclosure is negative.

Sample size	No sample-size calculations were performed. The number of replicates used for genetic screens (n = 2) is standard in the field, and data are presented as both individual and averaged values. Sample sizes in all experiments were determined to be sufficient due to effect size, level of variation within groups, consistency of measurable differences among groups, and statistical indicators of reproducibility.
Data exclusions	No data were excluded.
Replication	All experiments were confirmed with independent biological replicates, which demonstrated reproducible results. All attempts at replication were successful. The number of replicates for each experiment is clearly presented in the corresponding figure legends. Reproducibility in genetic screens is demonstrated with Pearson correlation coefficients calculated between replicate screens. Reproducibility of findings (and statistical noise) in other experiments can be assessed by comparing reported measures of variation (e.g. standard deviation) to reported measures of central tendency (e.g. medians/means), by examining the spread of individual data points and distributions that are visualized in figures, and by analyzing the results of orthogonal verification experiments that suggest similar findings. Additionally, a number of quality control measures were performed for more complex experiments--see Methods.
Randomization	Random allocation is not relevant to this study. Technically, experimental steps involving mixing (e.g. mixing of cells from a culture dish prior to measurement via flow cytometry) served to randomize which cells within an experiment were sampled and eliminate patterns that could arise from environmental covariates (e.g. differences due to proximity to the edge of a culture dish, differences due to proximity to the heating source or gas inlet in an incubator, etc).
Blinding	Investigators were not blinded to group allocation during data collection or analysis. Blinding was not relevant to this study because assignment of data values to samples was automated (e.g. flow cytometry method programs) and not based on subjective investigator assessments or assigning values from an analog measurement device (e.g. tumor size measurement or histological grading). Blinding would not have been possible because the authors designing individual experiments also carried those experiments out due to superior expertise in given techniques.

Reporting for specific materials, systems and methods

We require information from authors about some types of materials, experimental systems and methods used in many studies. Here, indicate whether each material, system or method listed is relevant to your study. If you are not sure if a list item applies to your research, read the appropriate section before selecting a response.

Materials & experimental systems

n/a	Involved in the study
☐	☒ Antibodies
☐	☒ Eukaryotic cell lines
☒	☐ Palaeontology and archaeology
☒	☐ Animals and other organisms
☒	☐ Human research participants
☒	☐ Clinical data
☒	☐ Dual use research of concern

Methods

n/a	Involved in the study
☒	☐ ChIP-seq
☒	☐ Flow cytometry
☒	☐ MRI-based neuroimaging

Antibodies

Antibodies used

Primary antibodies used: anti-FLAG (Sigma-Aldrich, #F1804), anti-Lamin B1 (Abcam, #16048), and anti-α-Tubulin (Sigma-Aldrich, #T6199). Secondary antibodies used: anti-Rabbit IgG (Licor, #926-3221), anti-Mouse IgG (Life Technologies, #A21058). Dilutions are specified in Methods.

Validation

All antibodies employed in this study are validated reagents that are commercially available from reliable vendors used for the purposes for which they were designed according to manufacturer protocols. Manufacturer validation below:

For anti-FLAG (Sigma-Aldrich, #F1804): clear appearance, colorless, liquid, concentration = 1.0-1.2 mg protein/mL by UV absorbance, ELISA titer >= 50,000, specificity = detects a single band of protein on a Western Blot from mammalian crude cell lysates by chemiluminescent probing, sensitivity = detects 2 ng of FLAG-BAP fusion protein by Dot Blot using Chemiluminescent Detection, protease-free, electrophoresis = two major bands with purity >90% when analyzed by microfluidic gel capillary electrophoresis. See certificate of analysis at (https://www.sigmaaldrich.com/catalog/product/sigma/f1804?lang=en&region=US) for details.

For anti-Lamin B1 (Abcam, #16048): by Western Blot, ab16048 was shown to specifically react with LMNB1 (Lamin B1) in wild type HAP1 cells. No band was observed when LMNB1 (Lamin B1) knockout samples were used; additional manufacturer validation by immunohistochemistry. See (https://www.abcam.com/lamin-b1-antibody-nuclear-envelope-marker-ab16048.html) for complete certificate of analysis.

For anti-α-Tubulin (Sigma-Aldrich, #T6199): clear appearance, colorless, liquid, concentration = 1.0-1.2 mg protein/mL by UV absorbance, manufacturer validation by Western Blot, manufacturer validation for specificity. See (https://www.sigmaaldrich.com/catalog/product/sigma/t6199?lang=en&region=US) for complete certificate of analysis.

For anti-Rabbit IgG (Licor, #926-3221): Isolation of specific antibodies was accomplished by affinity chromatography using pooled rabbit IgG covalently linked to agarose. Based on ELISA and flow cytometry, this antibody reacts with the heavy and light chains of rabbit IgG, and with the light chains of rabbit IgM and IgA. This antibody was tested by dot blot and and/or solid-phase adsorbed for minimal cross-reactivity with human, mouse, rat, sheep, and chicken serum proteins, but may cross-react with immunoglobulins from other species. The conjugate has been specifically tested and qualified for Western blot and In-Cell Western™ assay applications. See (https://www.licor.com/bio/reagents/irdye-800cw-goat-anti-rabbit-igg-secondary-antibody) for complete details.

For anti-Mouse IgG (Life Technologies, #A21058): specificity validated by manufacturer via Western Blot. See (https://www.thermofisher.com/antibody/product/Goat-anti-Mouse-IgG-H-L-Highly-Cross-Adsorbed-Secondary-Antibody-Polyclonal/A-21058) for complete purity and specificity details.

Eukaryotic cell lines

Policy information about cell lines

Cell line source(s)

The murine Mll-Af9/NrasG12D acute myeloid leukemia line (RN2) was generated by the laboratories of Christopher Vakoc (CSHL) and Scott Lowe (MSKCC) as described previously (Zuber, J et al. Nature biotechnology 29, 79-83, 2011). The RN2 cell lines used in this study were a gift from the Vakoc laboratory. All other cell lines were obtained commercially as follows: K-562 (ATCC, CCL-243), HEK 293T (ATCC, CRL-3216), NIH3T3 (ATCC, CRL-1658), B16-F10 (ATCC, CRL-6475), MOLM-13 (DSMZ, ACC-554), HEL (ATCC, TIB-180), A549 (ATCC, CCL-185).

Authentication

Cell lines were authenticated by SNP profiling.

Mycoplasma contamination

All cell lines routinely tested Mycoplasma negative.

Commonly misidentified lines (See ICLAC register)

No cell lines used in the study are in this database.
