# Supplementary material for: High-performance CRISPR-Cas12a genome editing for combinatorial genetic screening
Source: Nat Commun. 2020 Jul 13;11:3455. doi: 10.1038/s41467-020-17209-1 (PMC7359328; doi:10.1038/s41467-020-17209-1)
Supplement: Supplementary file 10 — Description of Additional Supplementary Files [file 41467_2020_17209_MOESM10_ESM.pdf]

Title: Supplementary Data 1
Description: crRNA sequences for competition based proliferation assays. List includes 23 nt spacer sequences for Fig. 1c-e, Supplementary Fig. 4b-c, Supplementary Fig. 5, Supplementary Fig. 6, Supplementary Fig. 12, and Supplementary Fig. 13. 'e' denotes targeting within the specified exon number.

Title: Supplementary Data 2
Description: crRNA included in tiling library to establish library design principles. List includes 23 nt spacer sequences for the crRNA in the tiling library used in Fig. 2b-c, Supplementary Fig. 7a-b. and Supplementary Fig. 8. The library contains 2298 different crRNA. DNA sequence target is noted within the crRNA name, and general region of targeting (protein domain, coding region non-domain, or non-coding) is specified.

Title: Supplementary Data 3
Description: crRNA included in the single level epigenetic dropout screen library. List includes 23 nt spacer sequences for the crRNA in the single-crRNA library targeting murine epigenetic factors used in Fig. 2d-e, Supplementary Fig. 9a-b, and Supplementary Fig. 10a-c. The library contains 787 different crRNA targeting domain regions (or non-domain regions for negative controls). DNA sequence target domain is noted within the crRNA name. 'e' denotes targeting within the specified exon number.

Title: Supplementary Data 4
Description: crRNA included in the pairwise epigenetic dropout screen library. List includes 23 nt spacer sequences for the crRNA in the dual-crRNA library targeting murine epigenetic factors used in Fig. 3b. The library contains 91 different crRNA targeting domain regions (or non-domain regions for negative controls), and contains 8281 pairwise combinations (includes positive and negative controls). DNA sequence target domain is noted within the crRNA name. 'e' denotes targeting within the specified exon number. Individual dual-crRNA sequences for validation experiments in Fig. 3c-d and Supplementary Fig. 17 were obtained from this library.

Title: Supplementary Data 5
Description: Primer sequences for all CRISPR library preparation. Custom barcodes were used for the mapping and de-multiplexing of pooled CRISPR library samples. Short barcode sequences (7-10 nt) were combined with NGS adaptor sequences specific to either AsCas12a or SpCas9 for amplification of the CRISPR RNA cassette (1st Round PCR). Primers to introduce the Illumina sequencing adaptors are indicated (2nd Round PCR).

Title: Supplementary Data 6
Description: Gene signatures used for RNA-seq analysis. List includes genes that comprise either the Leukemia Stem Cell Signature (LSC) or the Myeloid Differentiation Signature, as described by [Somervaille et al., Cell Stem Cell (2009) vol. 4 (2) pp. 129-40)] and [Brown et al., J Leukoc Biol. 2006 Aug;80(2):433-47], respectively. Gene signatures used for Fig. 3e and Supp. Fig. 18.
