# Supplementary material for: High-performance CRISPR-Cas12a genome editing for combinatorial genetic screening
Source: Nat Commun. 2020 Jul 13;11:3455. doi: 10.1038/s41467-020-17209-1 (PMC7359328; doi:10.1038/s41467-020-17209-1)
Supplement: Supplementary file 9 — Peer Review File [file 41467_2020_17209_MOESM9_ESM.pdf]

Editorial Note: This manuscript has been previously reviewed at another journal that is not operating a transparent peer review scheme. This document only contains reviewer comments and rebuttal letters for versions considered at *Nature Communications*. Mentions of prior referee reports have been redacted.
